# Genomic Face-off: An *In Silico* Comparison of the Probiotic Potential of *Lactobacillus* spp. and *Akkermansia muciniphila*

**DOI:** 10.2174/0113892029317403240815044408

**Published:** 2024-08-23

**Authors:** Md. Fakruddin, Md. Abu Bakar karim, Bakhtiar Ul Islam, Ashrafus Safa, Jinath Sultana, Nayeema Bulbul, Md. Asaduzzaman Shishir

**Affiliations:** 1 Department of Biochemistry & Microbiology, North South University, Dhaka, Bangladesh;; 2 Department of Life Sciences, Independent University, Dhaka, Bangladesh;; 3 Department of Microbiology, Dhaka International University, Dhaka, Bangladesh

**Keywords:** *In silico*, genome, sequence, probiotic, *Akkermansia muciniphila*, *Lactobacillus*

## Abstract

**Introduction:**

The gut microbiota plays a crucial role in maintaining human health, and probiotics have gained significant attention for their potential benefits. Among the diverse array of gut bacteria, *Akkermansia muciniphila,* and *Lactobacillus* spp. have emerged as promising candidates for their putative probiotic properties.

**Methods:**

In this study, we conducted a comprehensive comparative *in silico* analysis of the genomes of *A. muciniphila* and *Lactobacillus* to decipher their probiotic potential. Utilizing a range of bioinformatics tools, we evaluated various genomic attributes, including functional gene content, metabolic pathways, antimicrobial peptide production, adhesion factors, and stress response elements. These findings revealed distinctive genomic signatures between the two genera. *A. muciniphila* genomes exhibited a high prevalence of mucin-degrading enzymes, suggesting a specialized adaptation for mucin utilization in the gut environment.

**Results:**

Additionally, the presence of specific pathways for short-chain fatty acid production highlighted its potential impact on host health. *Lactobacillus* genomes, on the other hand, demonstrated a diverse repertoire of functional genes associated with probiotic attributes, including the production of antimicrobial peptides and adhesion factors, indicating potential for host-microbe interactions and immune modulation. Furthermore, this analysis unveiled the genetic basis of stress tolerance in both genera, revealing conserved mechanisms for surviving the dynamic conditions of the gut ecosystem.

**Conclusion:**

This study also shed light on the distribution of antibiotic-resistance genes, allowing us to assess safety concerns associated with their potential use as probiotics. Overall, this comparative *in silico* exploration provides valuable insights into the genomic foundation of *A. muciniphila* and *Lactobacillus* probiotic potential. These findings contribute to the understanding of their respective roles within the gut microbiota and offer a foundation for further experimental investigations. As probiotic applications continue to expand, this study advances our knowledge of the genetic underpinnings that govern their functionality and highlights promising avenues for future therapeutic interventions and personalized health strategies.

## 1 INTRODUCTION

The human gastrointestinal tract serves as a complex and dynamic ecosystem inhabited by a diverse community of microorganisms collectively known as the gut microbiota [1, 2]. Emerging evidence has underscored the pivotal role of these microbes in maintaining host health, influencing various physiological processes, and shaping immune responses [3, 4]. Among the multitude of microorganisms residing in the gut, certain bacterial species have garnered considerable attention for their potential to confer health benefits upon their host [4-6]. These microorganisms, commonly referred to as probiotics, have sparked intense research efforts aimed at unraveling their molecular mechanisms and deciphering their therapeutic potential [7-9].

*Akkermansia muciniphila* and *Lactobacillus* are two genera of bacteria that have emerged as intriguing candidates for probiotic interventions [4, 10-13]. *A. muciniphila*, a mucin-degrading bacterium, has been associated with a plethora of health-promoting effects, including improved gut barrier function, metabolic homeostasis, and modulation of immune responses [11, 14, 15]. *Lactobacillus*, a diverse and versatile genus, has demonstrated probiotic attributes encompassing antimicrobial activity, immune modulation, and enhancement of gastrointestinal health [16-18].

While the biological significance of *A. muciniphila* and *Lactobacillus* in the context of probiotics is increasingly recognized, a comprehensive understanding of their probiotic potential at the genomic level remains incomplete. Genomic analysis serves as a powerful tool to decipher the genetic underpinnings of microbial traits and functionalities [19]. In this pursuit, *in silico* approaches have gained prominence, offering a cost-effective and efficient means to explore and compare the genomes of diverse microorganisms [20, 21].

This study presents a systematic and comparative *in silico* analysis of the genomes of *A. muciniphila* and *Lactobacillus* species. By scrutinizing their genomic content, metabolic pathways, functional genes, and stress response mechanisms, this study aims to unravel the molecular foundations that contribute to their probiotic attributes. Understanding and analyzing these metabolic & stress response mechanisms in probiotics is crucial for optimizing their functionality and efficacy in promoting gut health. By studying the specific stress response capabilities that enable probiotics to adapt to the challenging environment of the GI tract, more resilient and effective probiotic strains can be selected and further developed. The overarching goal is to decipher how these bacterial genera have evolved and adapted to their niches within the gut ecosystem, potentially leading to a more comprehensive understanding of their interactions with the host.

Furthermore, the comparative nature of this analysis seeks to highlight both shared and distinct genomic features between *A. muciniphila* and *Lactobacillus*, shedding light on their divergent probiotic potential and offering insights into their potential roles in gut health. Ultimately, the findings of this study contribute to the broader field of probiotic research by providing a deeper understanding of the genetic determinants underlying the beneficial effects attributed to *A. muciniphila* and *Lactobacillus*, thus paving the way for informed and targeted probiotic interventions for improved human health [22, 23].

## 2 MATERIALS AND METHODS

### 2.1 Data Collection and Genome Retrieval

Genomic sequences of *A. muciniphila* and *Lactobacillus* spp. were obtained from publicly available databases, including GenBank and RefSeq. A total of 20 sets of genomic data (Table **1**) were selected based on their relevance to probiotic applications and the availability of high-quality genome assemblies. A diverse set of strains from each genus was included to ensure comprehensive representation.

### 2.2 Genome Annotation and Functional Annotation

The retrieved genomic sequences were annotated using state-of-the-art genome annotation tools. Protein-coding genes were predicted, and their functions were annotated using the Prokaryotic Genome Annotation System (Prokka) v1.14.5 [24] and Rapid Annotation using Subsystem Technology (RAST) [25]. Additional analysis was implemented by the Kyoto Encyclopedia of Genes and Genomes (KEGG) (https://www.kegg.jp/blastkoala/) [26]. Furthermore, functional annotations included the identification of multi-locus sequence typing (MLST) [27], multiple sequence alignment by MUSCLE [28], large phylogenetic data analysis using RAxML v8 [29, 30], sub-system analysis [31], screening antimicrobial resistance or virulence genes using ABRICATE [32], pangenome analysis by ROARY [33], gene ontology by DAVID [34], and antimicrobial peptide genes by BAGEL4 [35].

### 2.3 Comparative Genomic Analysis

Comparative analysis was performed using bioinformatics tools to compare the genomes of *A. muciniphila* and *Lactobacillus*. Genomic features such as gene content, orthologous gene clusters, and synteny were analyzed using PROKKA, Clusters of Orthologous Groups (COGs) (http://eggnog-mapper.embl.de/) (E-value < 0.001) [36], MLST to identify shared and unique attributes between the two genera, and CIRCOS [37] and BRIG [38] to visualize the genomic data in a circular layout.

### 2.4 Metabolic Pathway Analysis

Metabolic pathways were reconstructed and analyzed using KEGG to assess the potential contributions of *A. muciniphila* and *Lactobacillus* to host health. Special emphasis was placed on pathways related to carbohydrate production, iron acquisition, regulation and cell signaling, and other vitamins and cofactors with relevance to probiotic functionality. The comparison was then illustrated using Heatmapper (http://heatmapper.ca/).

### 2.5 Antimicrobial Peptide Prediction

Candidate antimicrobial peptides were predicted using BAGEL4 and antiSMASH v7.0 (https://antismash.secondarymetabolites.org/) [39]. Genes encoding antimicrobial peptides were identified, and their sequence characteristics were analyzed to infer potential antimicrobial activity. A heatmap was used to illustrate the distribution of genes among *A. muciniphila* and *Lactobacillus* spp.

### 2.6 Stress Response Analysis

Stress response elements, including genes associated with oxidative stress, heat shock, and other environmental stressors, were identified using sub-system analysis [31] and compared between *A. muciniphila* and *Lactobacillus* genomes by illustrating a heatmap by Heatmapper (http://heatmapper.ca/).

### 2.7 Antibiotic Resistance Profiling

Proteins related to Antibiotic resistance were identified using specialized databases such as VFDB [40], NCBI AMRFinder [41], Resfinder [42], and CARD [43] tools using ABRICATE [32] to assess potential safety concerns associated with the use of *A. muciniphila* and *Lactobacillus* genomic data.

## 3 RESULTS

### 3.1 Taxonomy and Phylogenomic Analysis

**Fig. (1):**
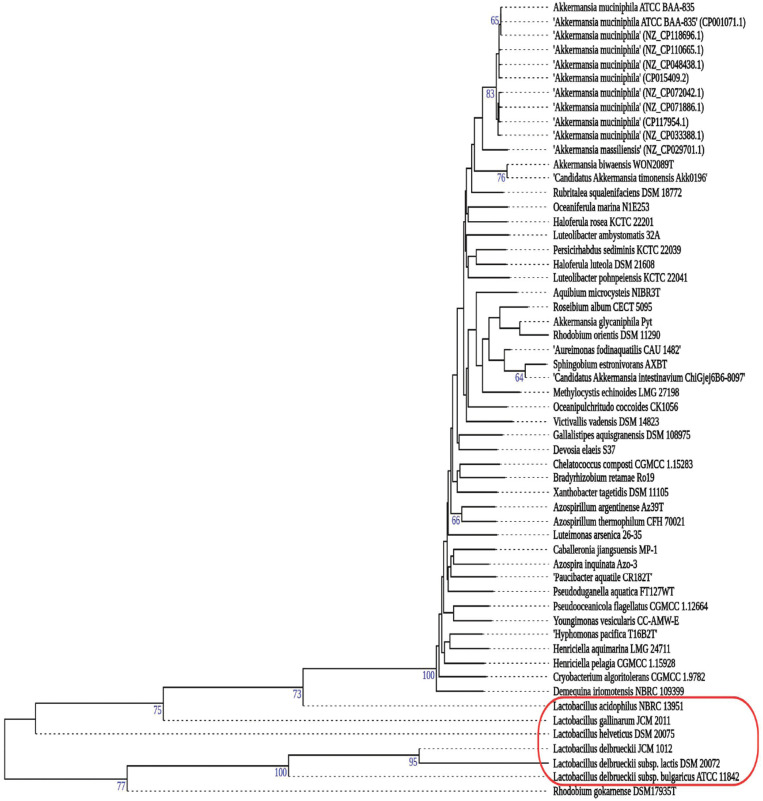
Taxonomic differences between *A. muciniphila* and *Lactobacillus* spp. The analysis showed a distant evolutionary relationship between *A. muciniphila* and *Lactobacillus* spp, with extensive branch lengths indicating genetic divergence. The genus's diversity and association with multiple outgroups suggested complex evolutionary interactions and potential adaptations to environmental niches.

The analysis conducted in Fig. (**1**) revealed a distant evolutionary relationship between *A. muciniphila* and *Lactobacillus* spp (Fig. **1**). The placement of these two genera on the same phylogenetic tree suggested a shared ancestry; however, the considerable branch lengths indicated a substantial evolutionary distance. The extensive branch lengths observed in the phylogenetic tree reflect a significant degree of genetic divergence or evolutionary time, indicating distinct evolutionary trajectories for *A. muciniphila* and *Lactobacillus* spp. Notably, the presence of numerous clades and subgroups within the same sub-branch as *A. muciniphila* highlighted the rich diversity within this genus. The consistently high bootstrap values at key nodes of the phylogenetic tree provided robust support for the observed relationships, reinforcing the confidence in the placement of *A. muciniphila* within the broader context of microbial evolution. Furthermore, the associations of *A. muciniphila* with multiple outgroups suggested complex evolutionary interactions and potential adaptations to various environmental niches. The analysis of genetic variation within *A. muciniphila* and *Lactobacillus* spp revealed a moderate level of diversity, suggesting a certain level of genetic stability within these groups. The placement of *A. muciniphila* within numerous clades and subgroups pointed at potential ecological specialization, emphasizing the need for further investigations into specific ecological niches and host-microbe interactions. Lastly, the taxonomic divergence at the Phylum level between *A. muciniphila* and *Lactobacillus* spp underscored the evolutionary divergence between these two genera, despite their cohabitation within the gut microbiome.

### 3.2 Comparison of Genomic Features

After annotation, we compared the 20 sets of genomic data. Table **2** presents a statistical summary of genome assembly and annotation for *A. muciniphila* and *Lactobacillus* spp. The dataset includes various genomic attributes such as base count, coding sequences (CDS), contigs, CRISPR elements, genes, ribosomal RNA (rRNA), transfer-messenger RNA (tmRNA), and transfer RNA (tRNA). The average size of the *A. muciniphila* genome was found to be 28,143,730 bases, with a standard deviation of 147,094.5 In contrast, the average genome size of *Lactobacillus* spp. was 2,451,495 bases, with a standard deviation of 466,650.5 The significant difference in genome size between these two genera suggested variations in genetic complexity and functional components. *A. muciniphila* has an average of 2,333 coding sequences, with a standard deviation of 122.70. On the other hand, *Lactobacillus* spp. has an average of 2,372 coding sequences, with a standard deviation of 428.33. The count of coding sequences provided insights into the genomes' propensity to encode proteins, thereby reflecting the range of functional components within each genus. Both *A. muciniphila* and *Lactobacillus* spp. exhibited an average of 1 contig, indicating that their genome assemblies are predominantly intact with minimal fragmentation. Additionally, both genera have an average of 2 CRISPR elements. Regarding gene count, *A. muciniphila* has an average of 2,397 genes, with a standard deviation of 122.7 On the other hand, *Lactobacillus* spp. has an average of 2,457 genes, with a standard deviation of 422.33. In terms of rRNA genes, *A. muciniphila* has an average of 9, while *Lactobacillus* spp. has an average of 17. The quantity of rRNA genes is crucial for understanding an organism's translational machinery and growth rate. Both genera possess a single tmRNA and a variable number of tRNA genes, which play important roles in protein synthesis and cellular function.

### 3.3 Genomic Plasticity Analysis

**Fig. (2):**
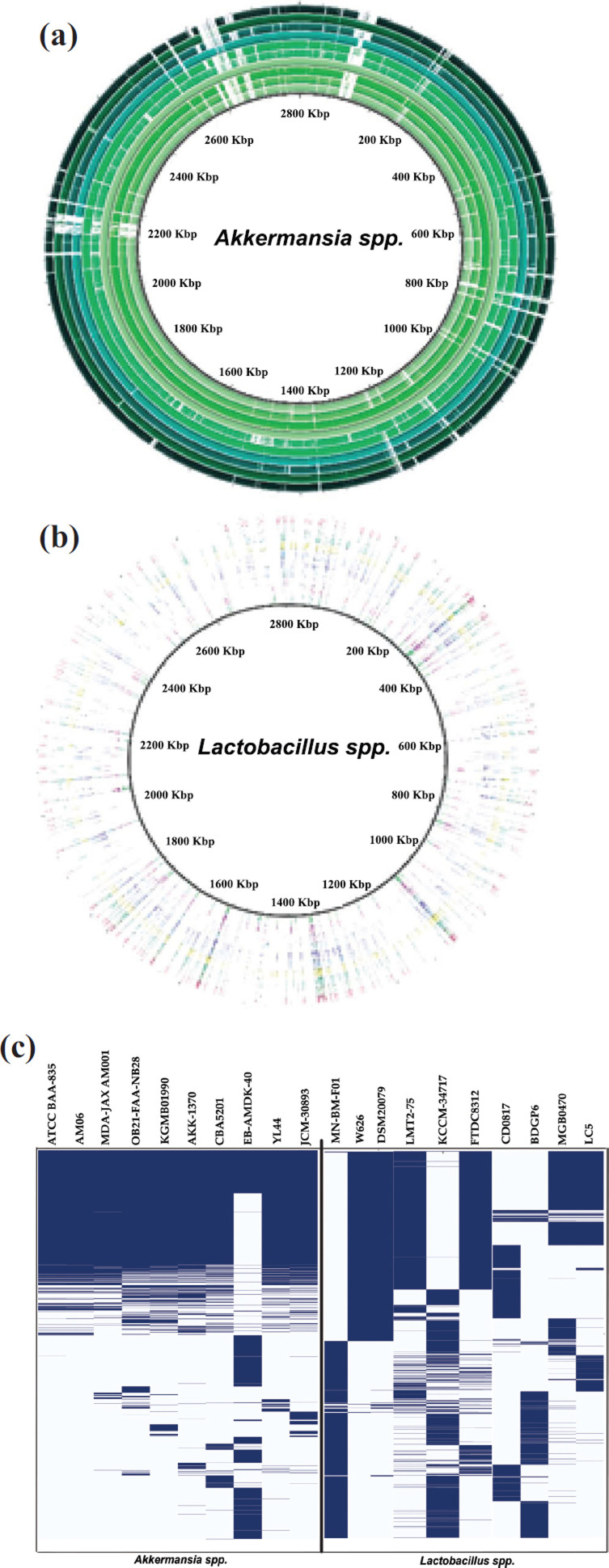
Genomic comparison between whole genomes of *A. muciniphila* and *Lactobacillus* spp. The BRIG analysis (**a** & **b**), to identify present and missing genes, compared *Lactobacillus* strains in the same clade, revealing higher unique gene frequencies than *A. muciniphila*. The pan-genome matrix (**c**) reveals shared genes between *A. muciniphila* and *Lactobacillus* strains, with 652 genes accounting for 11.08% of the pangenome. The accessory genome has 2149 shell genes and 3085 cloud genes. The *Lactobacillus* strains have a higher frequency of unique genes than *A. muciniphila*. For more details, see the supplementary file.

The pan-genome matrix (Fig. **2a-c**) gives an overview of genes that are shared between *A. muciniphila* and *Lactobacillus* strains. These indicate gene groupings present in different strain clusters. The generated matrix represents the presence and absence of core and accessory genes. The pangenome matrix identified a total of 5886 genes across all *A. muciniphila.* Among them, 652 genes were found to be core genes, accounting for 11.08% of the pangenome and shared by all *A. muciniphila.* Additionally, the accessory genome consisted of 2149 shell genes shared by less than 9 *A. muciniphila* isolates and 3085 cloud genes present in less than 10% of the isolates. Similarly, a pangenome analysis of *Lactobacillus* spp. was conducted, revealing varying numbers of core genes among different species and highlighting a high level of dissimilarity in their genetic makeup, except for a few loci. Among 5194 genes, less than 100 came out to be core materials and shared by all *Lactobacillus* spp. This comparison of *Lactobacillus* strains in the same clade (Fig. **2b**) highlighted that the strains have a higher frequency of unique genes than *A. muciniphila*. Another comparison to establish present and missing genes between the A. *muciniphila* strains and *Lactobacillus* strains was performed using BRIG (Fig. **2a** and **b**). More detailed genomic information of each strain has been provided in a **Supplementary file** protein synthesis and cellular functi.

### 3.4 Comparison of Antibiotic Resistance, Drug Target, and Transporters

*A. muciniphila* strains showcased a consistent and notable level of anti-microbial resistance (AMR) genes. In contrast, *Lactobacillus spp*. exhibited a wider range of AMR genes within the genus, spanning from 18 to 25. This observed discrepancy among strains implied varying selective pressures and potential discrepancies in susceptibility to antibiotics. Remarkably, the dataset unveiled an absence of drug target genes for all *A. muciniphila* strains. In terms of transporter genes, variability existed among the strains, ranging from 4 to 5. It is worth noting that a majority of the strains exhibited a higher count in this range, indicating a propensity for efficient transport mechanisms. The presence of varying transporter gene counts may underscore adaptive strategies within the *A. muciniphila* genus, potentially associated with their survival in the gut environment. In contrast, *Lactobacillus spp.* showcased a diverse array of drug transport genes and a broad range of transporter genes, spanning from 0 to 19. This heterogeneity within the genus suggested a broader spectrum of metabolic and transport strategies, aligning with the known diversity observed within *Lactobacillus* species. The observed variations in drug transport and transporter counts indicated variable abilities to respond to therapeutic compounds.

It is known that the presence of AMR-related genes (even full length) in a given genome does not directly imply antibiotic resistant phenotype. Hence, we utilized multiple packages such as AMRFinder, ResFinder, & Abricate to further elucidate antibiotic resistant phenotypes in the study genomes. Our analysis showed that none of the *Lactobacillus spp.* included in this study pose any antibiotic resistant phenotype. In the case of *A. muciniphila* strains, only two (EB AMDK 40 & KGMB01990) showed AMR phenotype against antibiotic, namely Erythromycin, Lincomycin, Clindamycin, Quinupristin, Pristinamycin IA & Virginiamycin S. Notably, none of the *A. muciniphila* and *Lactobacillus spp.* strains included in this study pose any plasmid, implicating that these strains, if used as probiotic bacteria will not be able to spread antibiotic-resistant phenotype both intra- and inter-specific strains (Table **3**).

### 3.5 Comparison of Probiotic Features

Analyzing these sub-system features in *A. muciniphila* and *Lactobacillus* spp. provides a comprehensive understanding of their functional capabilities, survival strategies, and beneficial impacts on the host's gut health. By assessing carbohydrate metabolism pathways, we can gain insights into how probiotics utilize dietary fibers, support gut health, and influence gut microbiota composition [44, 45]. Additionally, understanding the biosynthesis and uptake of cofactors and vitamins explains the nutritional contributions of probiotics to the host and their role in maintaining a balanced gut ecosystem [46]. Furthermore, studying the iron acquisition pathways helps us comprehend how probiotics sustain their growth and manage the iron balance in the gut, potentially impacting pathogen inhibition [47, 48]. Furthermore, identifying phages and prophages reveals insights into the evolutionary dynamics, potential for horizontal gene transfer, and stability of probiotic strains within the gut [49]. Apart from that, understanding regulatory systems and cell signaling pathways sheds light on how these probiotics respond to environmental signals, interact with the host, and maintain their functional capabilities under different gut conditions [50]. Finally, investigating virulence and defense mechanisms helps us understand how probiotics protect themselves, contribute to gut health, and prevent pathogenic infections [51, 52].

**Fig. (3):**
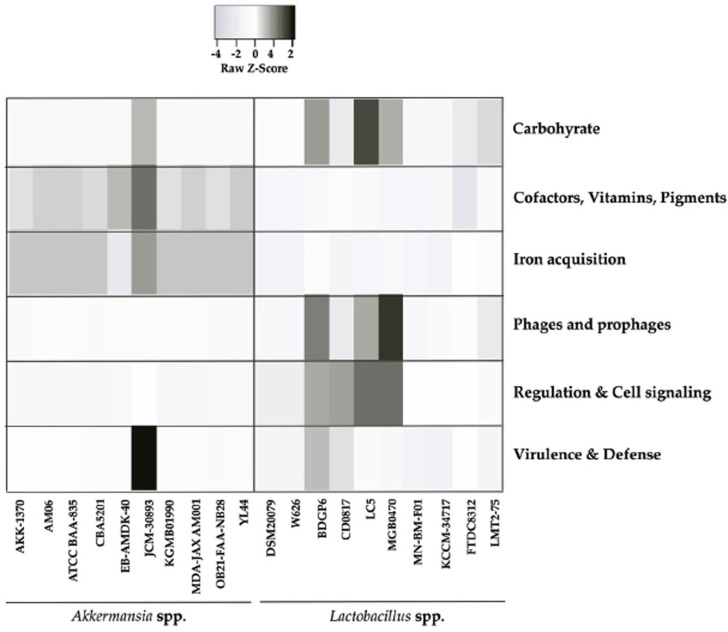
Host modulatory activity of *Lactobacillus* spp. and *A. muciniphila*. *Lactobacillus* spp. exhibit efficiency in glucose metabolism, while *A. muciniphila* strains have elevated scores in cofactors, vitamins, pigments, and iron acquisition subsystems. Clear differences may be observed in various aspects, such as phages, prophages, regulation, and cell signaling subsystems, with *Lactobacillus* spp. displaying higher scores. *A. muciniphila* strain JCM-30893 has significant scores in the virulence and defense subsystems, while *Lactobacillus* spp. provide more comprehensive data on their virulence systems.

The sub-system analysis (Fig. **3**) revealed that all strains of *A. muciniphila* exhibit low Z-scores, with the exception of strain JCM-30893. Conversely, within the *Lactobacillus* spp., strain LC5 demonstrated the highest Z-score, followed by strains BDGP6 and MGB0470, which both exhibited similar score levels. Overall, *Lactobacillus* spp. showed a prominent advantage in carbohydrate metabolism. In the subsystems related to cofactors, vitamins, pigments, and iron acquisition, all strains of *A. muciniphila* exhibited higher scores compared to the *Lactobacillus* spp. This indicates a potential for *A. muciniphila* to maintain a more balanced gut ecosystem in the host than the *Lactobacillus* spp., while sustaining itself and inhibiting pathogens. However, significant differences were observed in the scores for the phages and prophages, as well as in the regulation and cell signaling subsystems. None of the strains of *A. muciniphila* exhibited significant scores that provided insights into evolutionary dynamics in these areas. Nevertheless, the *Lactobacillus* spp. demonstrated higher scores in the phage and cell signaling subsystems. Specifically, strain MGB0470 had the highest scores, followed by strains BDGP6, LC5, and CD0817 in both subsystems. Finally, in the virulence and defense subsystem, only *A. muciniphila* strain JCM-30893 exhibited a significant score, whereas the *Lactobacillus* spp provided more substantial data to offer thorough insights into their virulence systems.

**Fig. (4):**
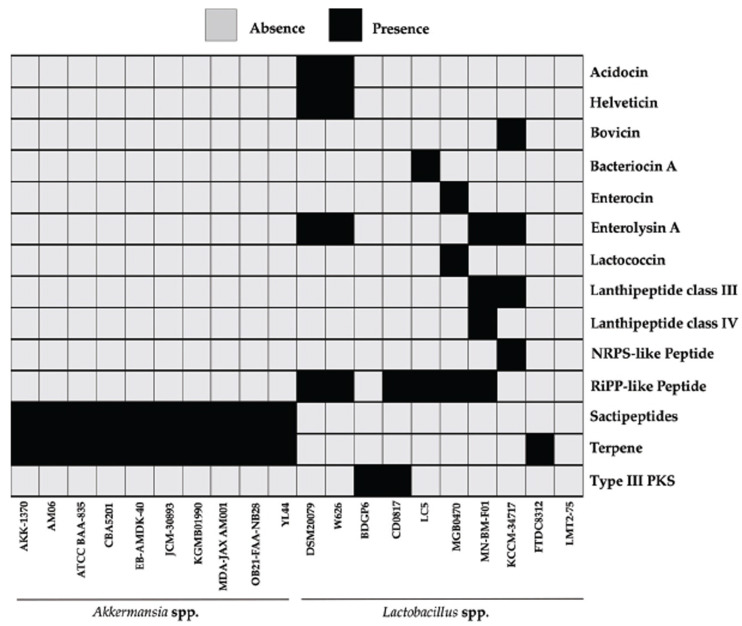
Comparison of the presence of probiotic peptides between *Lactobacillus* spp. and *A. muciniphila.* All strains of *A. muciniphila* exhibited the presence of Sactipeptides and Terpene, whereas only strain FTDC8312 of *Lactobacillus* showed such characteristics. The other strains showed a varied distribution of probiotic peptides.

The heatmap analysis (Fig. **4**) revealed the distribution of antimicrobial compounds among different strains of *A. muciniphila* and *Lactobacillus* spp.. Acidocin was not detected in any strains of *A. muciniphila*, while in *Lactobacillus* spp., it was only present in DSM20079 and W626 strains. Similarly, Helveticin exhibited a similar pattern, being absent in all strains of *A. muciniphila* and present only in DSM20079 and W626 strains of *Lactobacillus* spp.. Bovicin, bacteriocin A, enterocin, and lactococcin were all absent in *A. muciniphila*, while in *Lactobacillus* spp., they were present in KCCM-34717, LC5, MGB0470, and MN-BM-F01 strains, respectively. On the other hand, Enterolysin A was absent in all strains of *A. muciniphila*, but present in DSM20079, W626, MN-BM-F01, and KCCM-34717 strains of *Lactobacillus* spp.. Lanthipeptide class III was absent in all strains of *A. muciniphila*, while in *Lactobacillus* spp., it was only present in MN-BM-F01 and KCCM-34717 strains. Lanthipeptide class IV, NRPS-like peptide, and RiPP-like peptide were absent in all strains of *A. muciniphila*, while in *Lactobacillus* spp., they showed varying presence across multiple strains. Sactipeptides were detected in all strains of *A. muciniphila*, whereas in *Lactobacillus* spp., they were not found. Terpene presence was observed in all strains of *A. muciniphila*, while in *Lactobacillus* spp., it was only present in the FTDC8312 strain. Lastly, Type III PKS was absent in all strains of *A. muciniphila*, while in *Lactobacillus* spp., it was only present in BDGP6 and CD0817 strains.

**Fig. (5):**
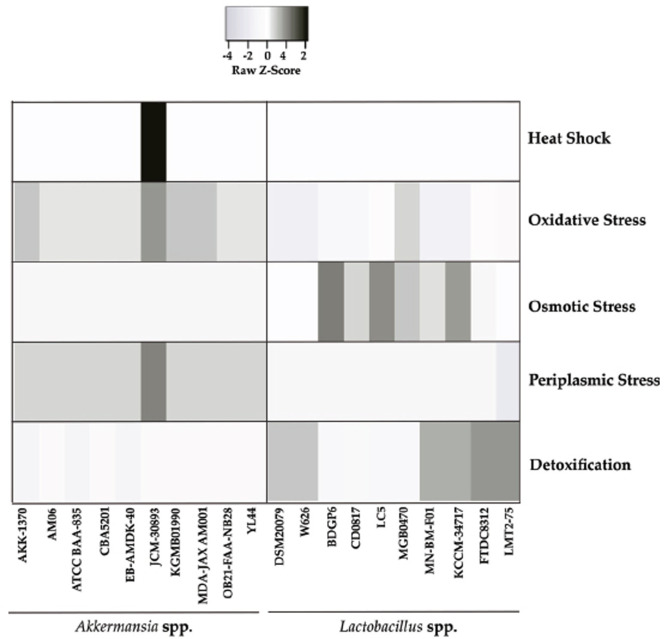
Comparison of stress resistance between *Lactobacillus* spp. and *A. muciniphila.* The stress response analysis revealed that *A. muciniphila* strain JCM-30893 exhibited a notable heat shock response. *A. muciniphila* strains displayed heightened oxidative and periplasmic stress responses in contrast to *Lactobacillus* spp., which predominantly exhibited elevated osmotic stress responses, barring certain strains.

The stress response analysis (Fig. **5**), conducted using the Pearson Distance Matrix, revealed distinct responses to various stress conditions in the examined *A. muciniphila* and *Lactobacillus* spp. strains. Notably, in *A. muciniphila*, only strain JCM-30893 exhibited a significant score in the heat shock response. Oxidative stress response was more pronounced in *A. muciniphila* strains compared to *Lactobacillus* spp. Conversely, the osmotic stress response was higher in *Lactobacillus* spp., with the exception of strains DSM20079, W65, FTDC8312, and LMT275. Furthermore, all strains of *A. muciniphila* demonstrated a superior response to periplasmic stress compared to *Lactobacillus* spp. Although *Lactobacillus* spp. showed a better detoxification score overall. Strain AKK 1370, ATCC BAA 835, and EB AMDK 40 of *A. muciniphila* exhibited somewhat significant scores in this category.

### 3.6 Comparison of Genomic and Metabolic Features

**Fig. (6):**
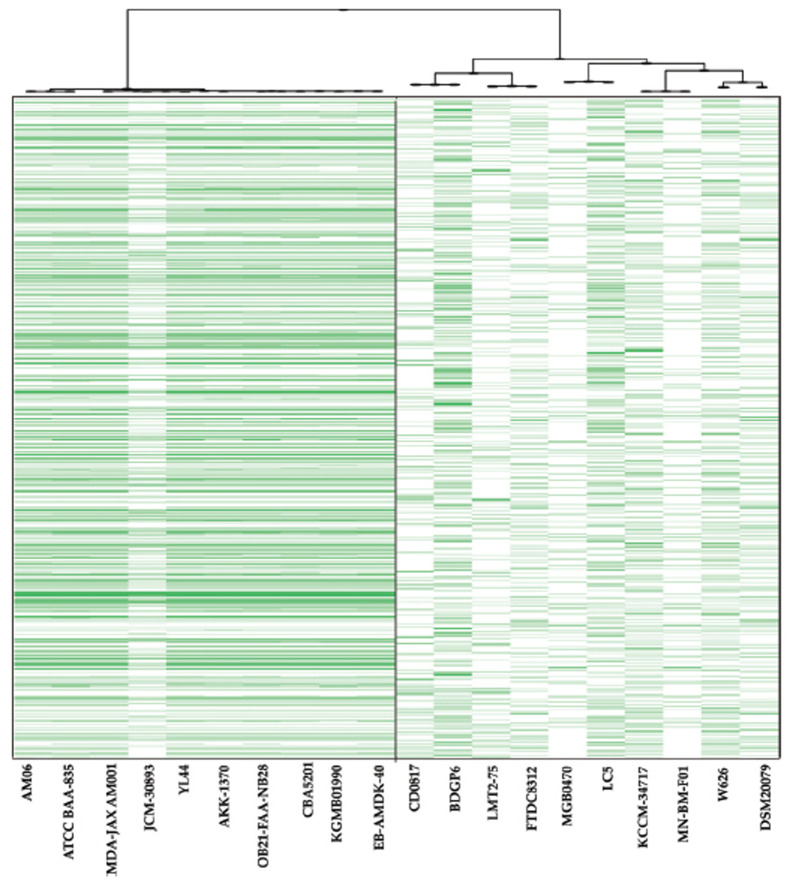
Genomic differences between *A. muciniphila* and *Lactobacillus* spp.. *A. muciniphila* and *Lactobacillus* spp. have distinct genome sizes and functional coordination. The strain JCM30893 of *A. muciniphila* strain has a focused range of functionality, allowing it to thrive in specific host environments. On the other hand, *Lactobacillus* spp.'s highly dispersed genomic regions enable interaction with a wider range of host molecules, potentially contributing to health benefits. These genomic and functional disparities shape each species' ability to colonize and thrive in the gut environment, potentially impacting disease processes.

*A. muciniphila* demonstrates significant variation in genome size, with the JCM30893 strain exhibiting a smaller genome (Fig. **6**). This analysis suggested a potential for a more concise range of functionality and adaptability in diverse host environments. In contrast, *Lactobacillus* spp. exhibited highly dispersed regions within its genome. This genomic arrangement enables *Lactobacillus* spp. to engage with a wider spectrum of molecules within the host system. Furthermore, *A. muciniphila* manifested a preference for clustered regions in its genome, indicating a high degree of functional coordination. This coordination potentially contributes to unique metabolic pathways, further augmenting the capabilities of *A.* muciniphila genomes.

The variations in genome size and organization significantly influence the functionality and interaction of both *A. muciniphila* and *Lactobacillus* spp. with the host. The smaller genome size of the JCM30893 strain of *A. muciniphila* implied a more focused range of functionality and adaptability. This specialization allows *A. muciniphila* to thrive in specific host environments and fulfill a distinct role in gut health. Conversely, the widely dispersed genomic regions of *Lactobacillus* spp. facilitated interaction with a broader array of host molecules. This diversity of interactions potentially contributes to a wide range of health benefits associated with *Lactobacillus* spp. These genomic and functional disparities shape the ability of each species to colonize and flourish in the gut environment. These probiotics also influence their roles in supporting host health and may have implications for their involvement in disease processes.

**Fig. (7):**
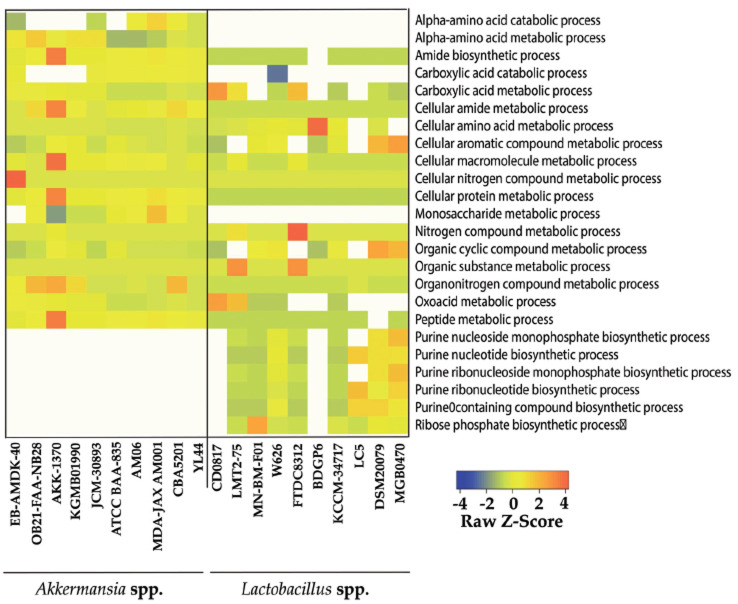
Metabolic differences between *A.* muciniphila and *Lactobacillus* spp. The study analyzed metabolic interactions between *A. muciniphila* and *Lactobacillus* spp., revealing a clustered metabolic region in *A. muciniphila*, and significant differences among all *Lactobacillus* spp. strains, with strains CD0817 and BDGP6 showing significant variation from traditional strains.

The analysis of metabolic interactions between *A. muciniphila* and *Lactobacillus* spp. is of particular significance in the fields of ecology, medicine, and biotechnology (Fig. **7**). Notably, all *A. muciniphila* strains exhibited an evident absence of purine-related biosynthetic processes. Conversely, all *Lactobacillus* spp. strains were devoid of alpha-amino acid metabolic and catabolic processes, as well as monosaccharide metabolic processes. It is also noteworthy that almost all *A. muciniphila* strains exhibited carboxylic acid catabolic processes, whereas only the W626 strain of *Lactobacillus* demonstrated a significantly low Z-score for this process. Overall, a clustered metabolic region was identified in *A. muciniphila*, while substantial differences were observed among all *Lactobacillus* spp. Note that, *Lactobacillus* strain CD0817 and BDGP6 demonstrated considerable variation from the traditional strains.

## 4 DISCUSSION

The gut microbiota's role in human health and disease has ignited a surge of interest in probiotics, and *A. muciniphila* and *Lactobacillus* spp. have emerged as intriguing candidates [12, 15, 23, 53]. In this study, a comprehensive comparative *in silico* analysis of the genomes of *A. muciniphila* and *Lactobacillus* to decode their probiotic potential and shed light on their genetic underpinnings was conducted. These findings provide valuable insights into the genomic foundations of these genera's putative health-promoting attributes and underscore their unique roles within the gut ecosystem.

This analysis unveiled distinct genomic signatures underlying the probiotic attributes of *A. muciniphila* and *Lactobacillus*. *A. muciniphila* exhibited a remarkable adaptation for mucin degradation, evidenced by the prevalence of mucin-degrading enzymes across its genomes [10, 11, 14, 54]. This specialization underscores its ability to interact with the host's mucosal layer, potentially enhancing gut barrier function and nutrient utilization [55-57]. Additionally, *A. muciniphila* genomes demonstrated enrichment in pathways related to short-chain fatty acid production, suggesting its potential to contribute to host metabolic health [58-60]. Conversely, *Lactobacillus* genomes featured a diverse repertoire of functional genes associated with probiotic attributes. The presence of antimicrobial peptide genes and adhesion factors highlights its potential for interactions with other gut inhabitants and modulation of immune responses [4, 61, 62]. The genetic basis for stress tolerance observed in both genera underscores their resilience within the dynamic gut environment [11, 55, 61-64].

The comparative analysis between *A. muciniphila* and *Lactobacillus* revealed shared and distinctive genomic features. While both genera demonstrated stress response mechanisms, the specific genes involved in these pathways varied, suggesting unique adaptations to environmental challenges. Furthermore, the distribution of antibiotic-resistance genes warrants careful consideration in their application as probiotics, emphasizing the importance of safety assessments [65-68].

The findings of this study hold significant implications for probiotic applications. The unique mucin-degrading capacity of *A. muciniphila* underscores its potential for supporting gut health and host-microbe interactions [10, 12, 69]. Meanwhile, *Lactobacillus* species showcase multifaceted attributes, including antimicrobial peptide production and adhesion factors, supporting their role in immune modulation and intestinal homeostasis. These insights could guide the selection and optimization of probiotic strains for targeted therapeutic interventions [12, 70, 71].

The *in-silico* analysis conducted in this study provides a foundational understanding of the genomic characteristics of *A. muciniphila* and *Lactobacillus* spp. However, it is essential to extend and validate these findings through experimental research [72-74]. Specifically, functional studies, including both *in vitro* and *in vivo* assays, are necessary to confirm the predicted probiotic attributes and interpret the mechanistic basis of the observed genomic features [75-78]. Such experimental validation will aid in translating genomic insights into practical applications. Moreover, the acquisition and analysis of additional high-quality genome sequences will significantly enhance the precision of comparative genomic analyses. This will not only extend our understanding of the genomic diversity and functional capabilities within these genera but also facilitate the identification of novel probiotic strains with optimized therapeutic potential [79]. Continued research in this area is critical for advancing the field of probiotics and developing innovative strategies to promote human health through microbiome modulation.

## CONCLUSION

This study reveals unique genetic signatures in each genus, illuminating their distinct roles within the gut microbiota. By uncovering specialized attributes such as mucin degradation and short-chain fatty acid production in *A. muciniphila*, and antimicrobial peptide production and adhesion factors in *Lactobacillus spp.*, the research provides valuable insights into their mechanisms of action. This knowledge is pivotal for guiding the development of targeted probiotic interventions that promote gut health, immune modulation, and metabolic balance. Moreover, the study's identification of stress response mechanisms and antibiotic resistance genes underscores the need for responsible probiotic selection and safety assessments. Ultimately, this research paves the way for informed probiotic strategies that leverage the genetic insights of *A. muciniphila* and *Lactobacillus* spp. to enhance human well-being and advance personalized microbiome-based therapies.

## AUTHORS’ CONTRIBUTIONS

MF, SBI, and MAS contributed to conceptualization. MF, MABK, AS, and MAS wrote the original draft. MABK, SBI, JSJ, AS, NB wrote the review and helped in editing.

## Figures and Tables

**Table 1:** Selected strains of *A. muciniphila* and *Lactobacillus* spp.

***Akkermansia muciniphila***	***Lactobacillus* spp.**
*A. muciniphila* (AKK1370)	*L. acidophillus* (DSM20079)
*A. muciniphila* (AM06)	*L. acidophillus* (W626)
*A. muciniphila* (ATCC BAA-835)	*L. brevis* (BDGP6)
*A. muciniphila* (CBA5201)	*L. brevis* (CD0817)
*A. muciniphila* (EB-AMDK-40)	*L. casei* (LC5)
*A. muciniphila* (JCM 30893)	*L. casei* (MGB470)
*A. muciniphila* (KGMB-1990)	*L. delbruckii bulgaricus* (MN-BM-F01)
*A. muciniphila* (MDA-JAX AM01)	*L. delbruckii lactis* (KCCM 34717)
*A. muciniphila* (OB21 FAA NB28)	*L. fermentum* (FTDC8312)
*A. muciniphila* (YL44)	*L. fermentum* (LMT2-75)

**Table 2:** Statistical summary of assembly and annotation of genomes of *A. muciniphila* and *Lactobacillus* spp.

***-***	***A. muciniphila***		***Lactobacillus* spp. **	
***-***	**Value (mean)**	**St. Dev**	**Value (mean)**	**St. Dev**
*Bases*	28143730	147094.5	2451495	466650.5
*CDS*	2333	122.70	2372	428.33
*Contigs*	1	0	1	0
*CRISPR*	2	1.19	2	1.164
*Genes*	2397	122.7	2457	422.33
*rRNA*	9	0	17	6.19
*tmRNA*	1	0	1	0
*tRNA*	54	0.42	66	15.06

**Table 3:** Prediction of gene count related to antibiotic resistance, drug target, and transporter among the *A. muciniphila* and *Lactobacillus* spp.

***Akkermansia muciniphila***	**Gene Count**		
	**Antibiotic Resistance**	**Drug Target**	**Transporter**
Akk1370	26	0	5
AM06	26	0	5
ATCC BAA0835	26	0	5
CBA5201	26	0	5
EB AMDK 40	27	0	4
JCM 30893	26	0	5
KGMB 1990	27	0	5
MDA JAX AM 01	26	0	5
OB21 FAA NB 28	26	0	5
YL44	26	0	5
***Lactobacillus* spp.**	**Gene Count**		
	**Antibiotic Resistance**	**Drug Target**	**Transporter**
DSM20079	19	2	12
W626	19	2	13
BDGP6	25	1	11
CD0817	24	0	5
LC5	23	5	18
MGB470	21	5	19
MN-BM-F01	18	4	3
KCCM 34717	19	5	2
FTDC8312	23	0	2
LMT2075	24	0	2

## Data Availability

The authors confirm that the data supporting the findings of this research are available within the article.

## LIST OF ABBREVIATIONS

AMR: Anti-microbial Resistance
COGs: Clusters of Orthologous Groups
KEGG: Kyoto Encyclopedia of Genes and Genomes
MLST: Multi-locus Sequence Typing
RAST: Rapid Annotation using Subsystem Technology
rRNA: ribosomal RNA
tmRNA: transfer-messenger RNA
tRNA: transfer RNA
