# Supplementary material for: Genomic Face-off: An In Silico Comparison of the Probiotic Potential of Lactobacillus spp. and Akkermansia muciniphila
Source: Curr Genomics. 2024 Aug 23;26(2):129–43. doi: 10.2174/0113892029317403240815044408 (PMC12105293; doi:10.2174/0113892029317403240815044408)
Supplement: Supplementary file 1 — Supplementary material is available on the publisher’s website along with the published article. [file CG-26-2-129_SD1.pdf]

Genomic Face-Off: An *In Silico* Comparison of the Probiotic Potential of *Lactobacillus* spp. and *Akkermansia muciniphila*

Supplementary Data:

Table S1: Statistical summary of assembly and annotation of genomes of *Akkermansia* spp.

	Akk1370	AM06	ATCC BAA0835	CBA5201	EB0AMDK040	JCM 30893	KGMB01990	MDA0JAX AM001	OB21 FAA NB 28	YL44
**Origin**	Human (USA)	Human (China)	-	Human (South Korea)	Human (South Korea)	Human (Japan)	Human (South Korea)	USA	Human (USA)	Mouse (Switzerland)
**Bases**	2803677	2664112	2664102	2860407	3156483	2878261	2844062	2675865	2851480	2745278
**GC Content**	55.35	55.76	55.8	55.32	57.78	55.63	55.23	55.7	55.46	55.66
**CDS**	2325	2182	2184	2401	2558	2408	2363	2201	2426	2282
**Contigs**	1	1	1	1	1	1	1	1	1	1
**CRISPR**	2	1	1	3	4	0	3	2	3	2
**Genes**	2389	2246	2250	2465	2623	2473	2427	2265	2490	2346
**rRNA**	9	9	9	9	9	9	9	9	9	9
**tmRNA**	1	1	1	1	1	1	1	1	1	1
**tRNA**	54	54	54	54	55	55	54	54	54	54
**Accession No.**	CP072042	CP118696	CP001071	CP033388	CP029702	CP048438	CP071886	CP110665	CP117954	CP015409

Table S2: Statistical summary of assembly and annotation of genomes of *Lactobacillus* spp.

	DSM20079	W626	BDGP6	CD0817	LC5	MGB0470	MN-BM-F01	KCCM 34717	FTDC8312	LMT2075
**Origin**	Human (France)	China	USA	China	Human (South Korea)	South Korea	China	-	Human (Malaysia)	South Korea
**Bases**	2009973	1978926	2785111	2990570	3132867	2940907	1875071	2263382	2239921	2298221
**GC Content**	34.71	34.72	45.57	50.34	47.92	47.9	49.71	49.08	50.1	50.49
**CDS**	1917	1880	2801	2769	2912	2891	1901	2169	2204	2281
**Contigs**	1	1	1	1	1	1	1	1	1	1
**CRISPR**	1	1	1	5	0	0	1	1	2	4
**Genes**	1990	1953	2888	2836	2988	2966	2014	2300	2279	2356
**rRNA**	12	12	15	16	15	15	24	32	15	15
**tmRNA**	1	1	1	1	1	1	1	1	1	1
**tRNA**	60	60	71	50	60	59	88	98	59	59
**Accession No.**	CP020620	CP128490	CP024635	CP032931	CP017065	CP064303	CP013610	CP018215	CP021104	CP034099

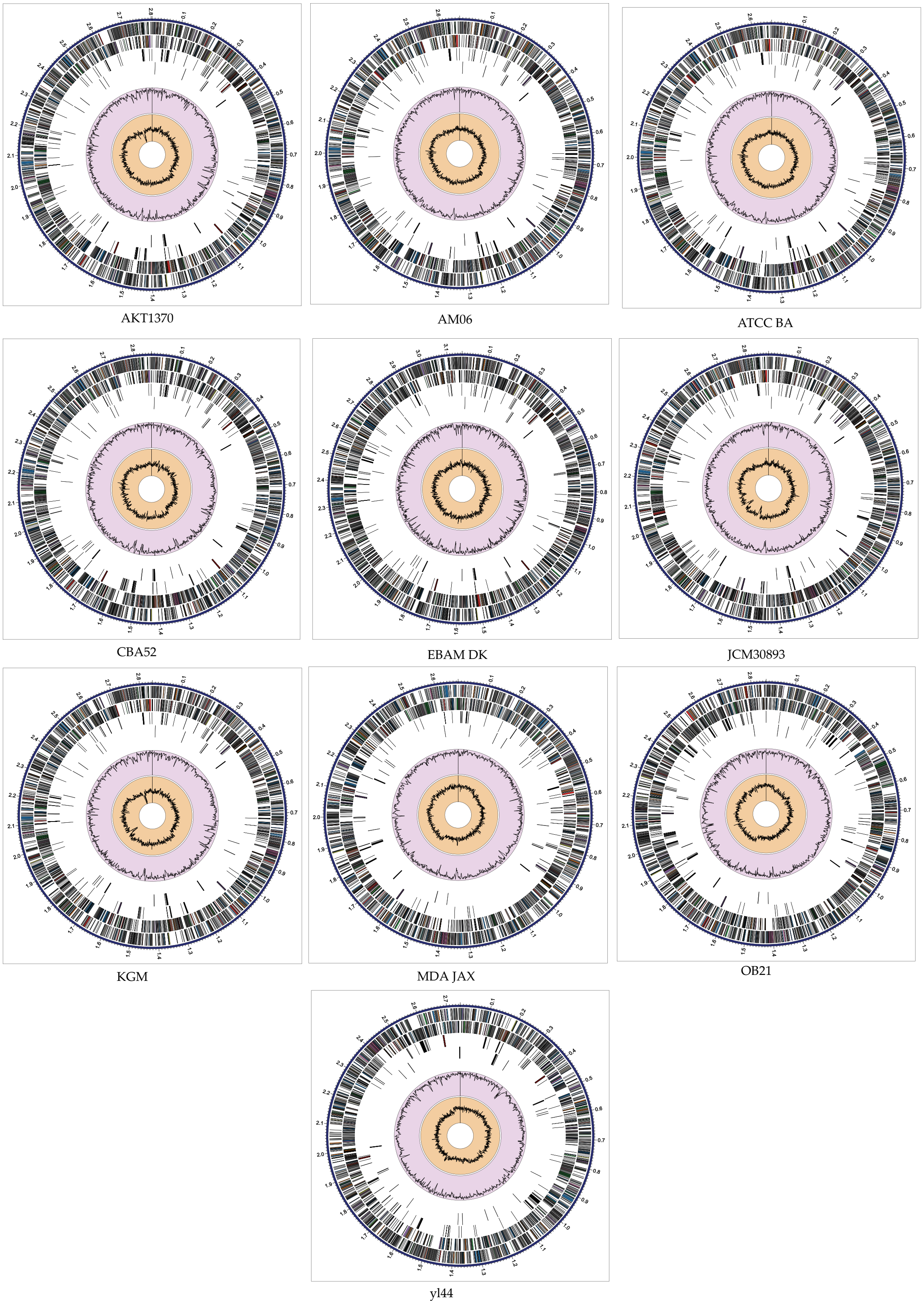

Supplementary Figure-1: Circos map of *Akkermansia muciniphila* genomes

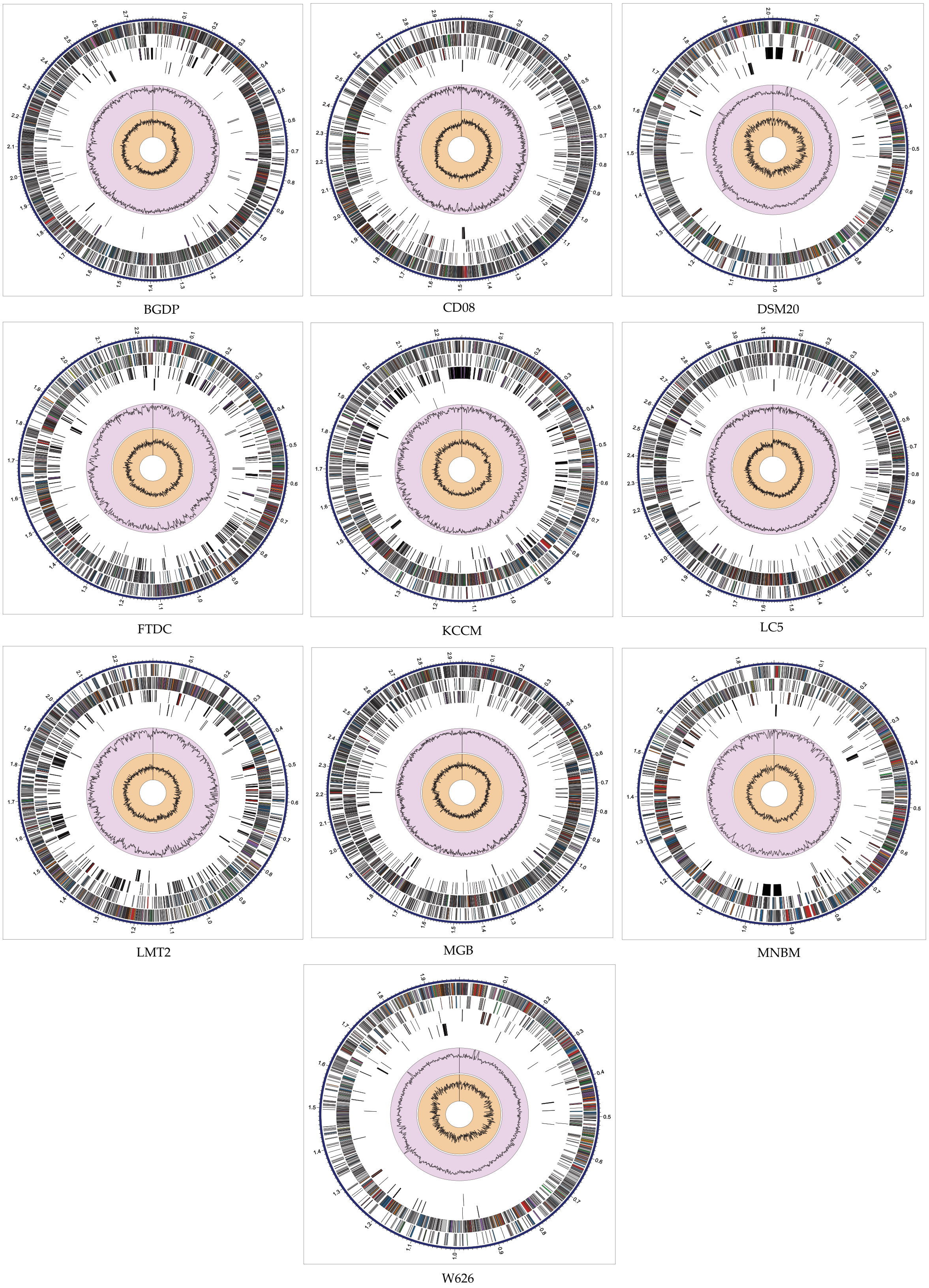

Supplementary Figure-1: Circos map of *Lactobacillus spp* genomes

Gene Ontology (GO) of *the Akkermansia muciniphila* and *Lactobacillus spp.*

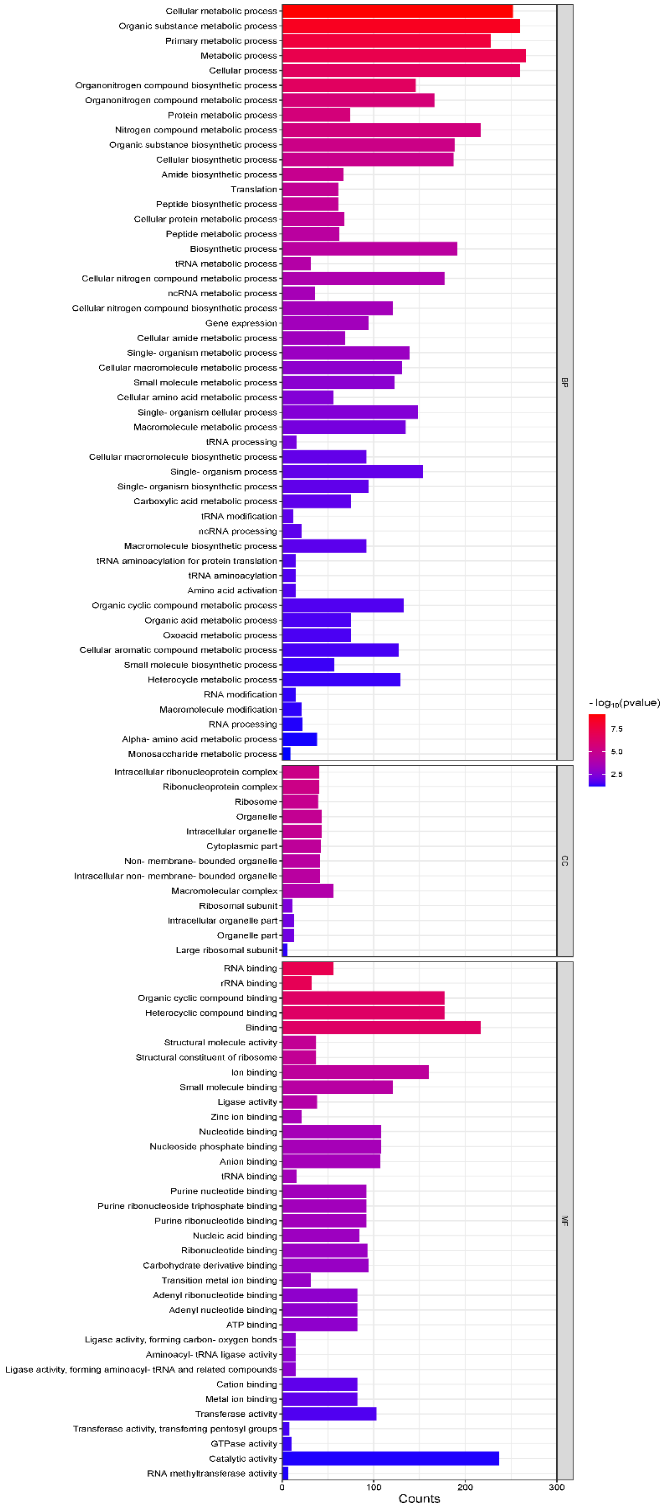

A. muciniphila AKK1370

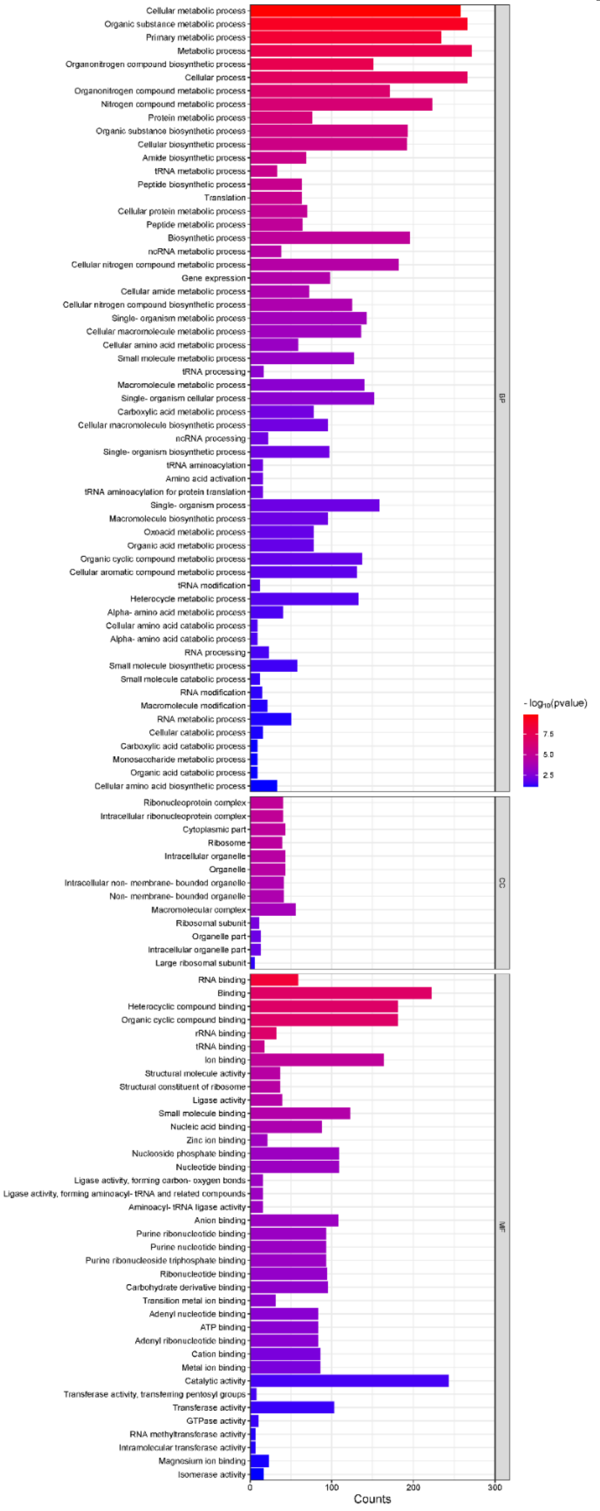

A. muciniphila AM06

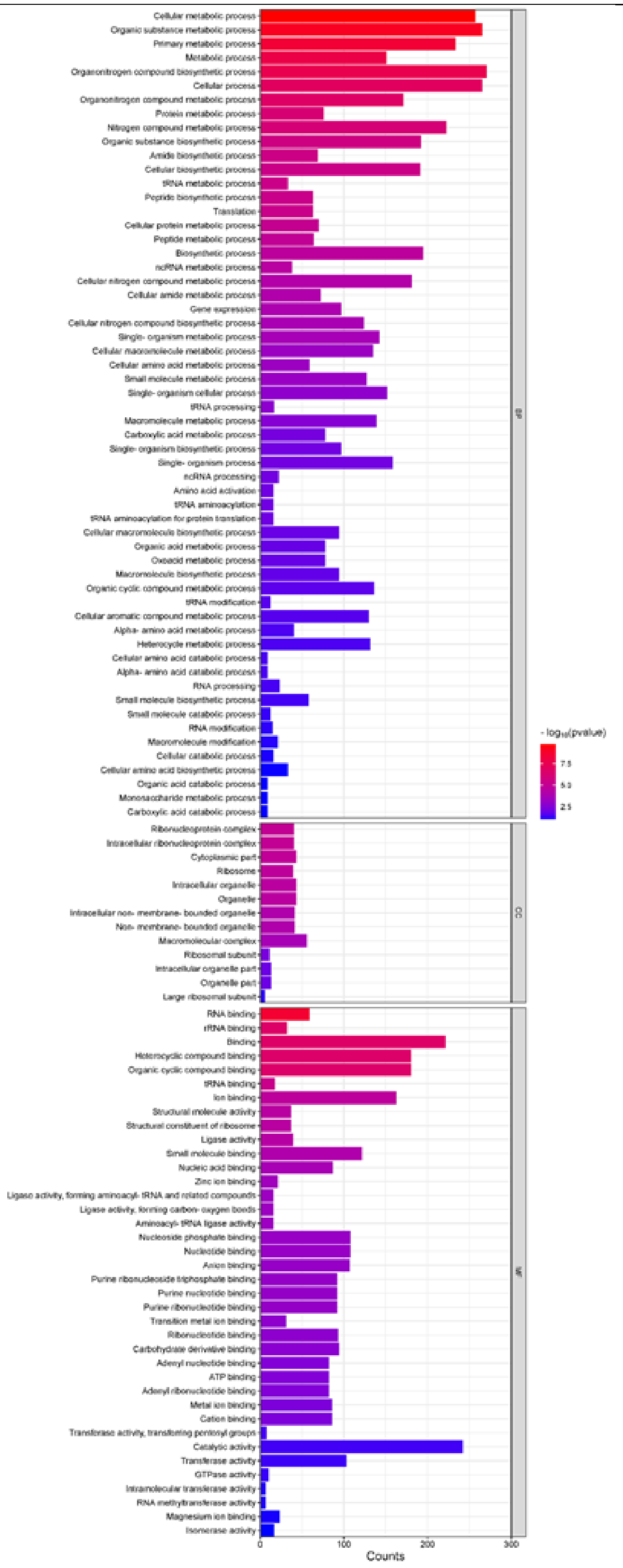

A. muciniphila ATCC BAA 835

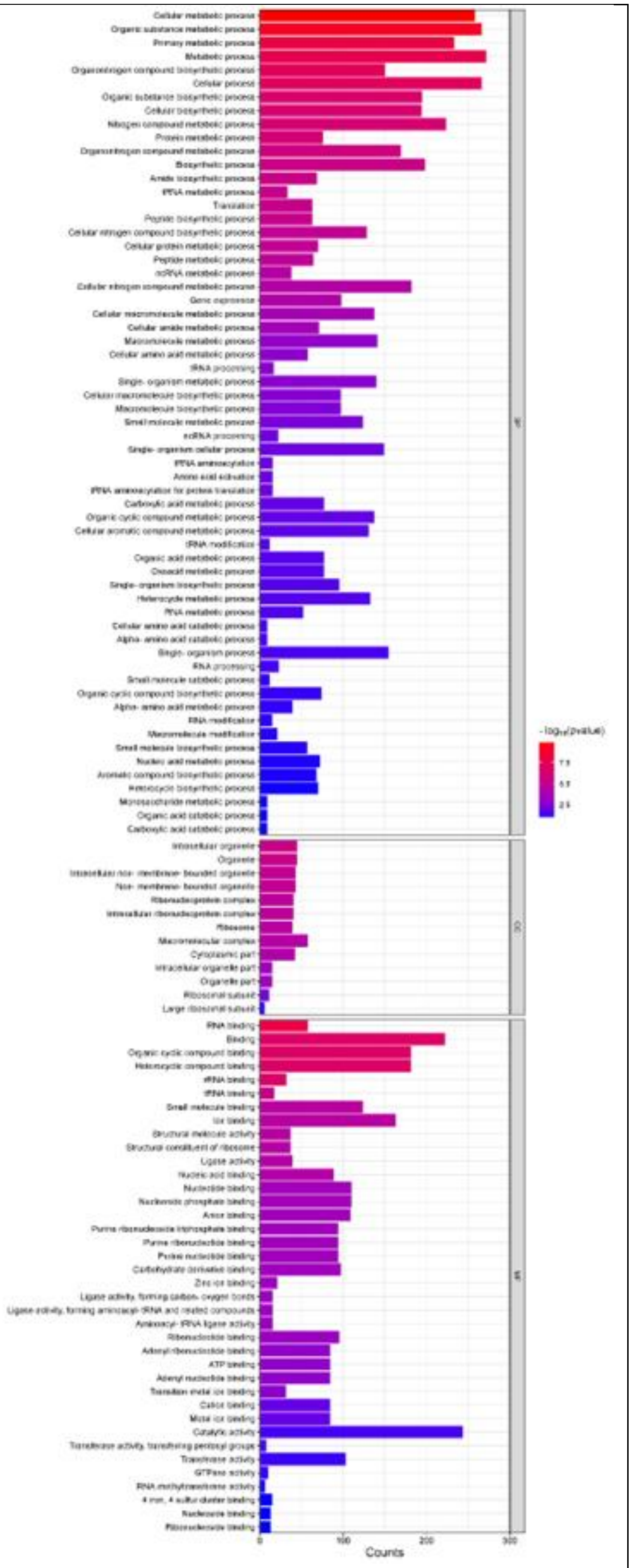

A. muciniphila CBA 5201

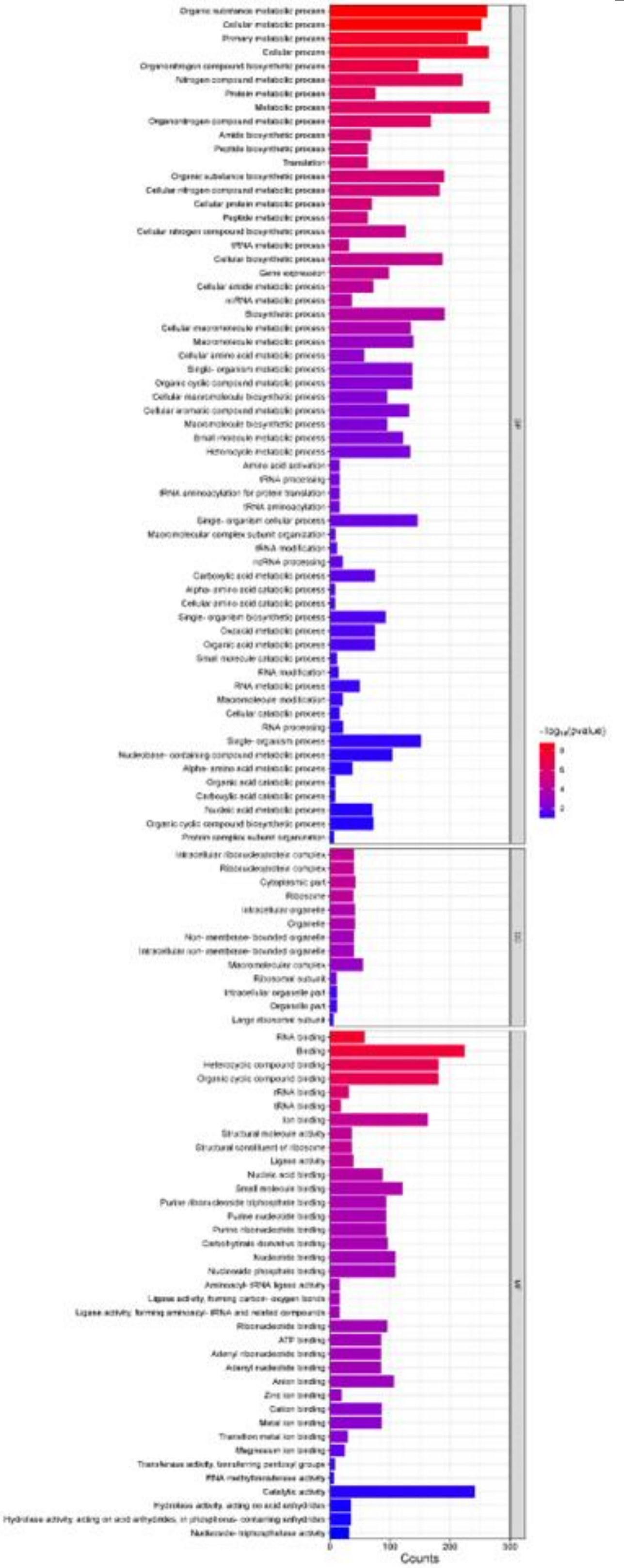

A. muciniphila EB AMDK 40

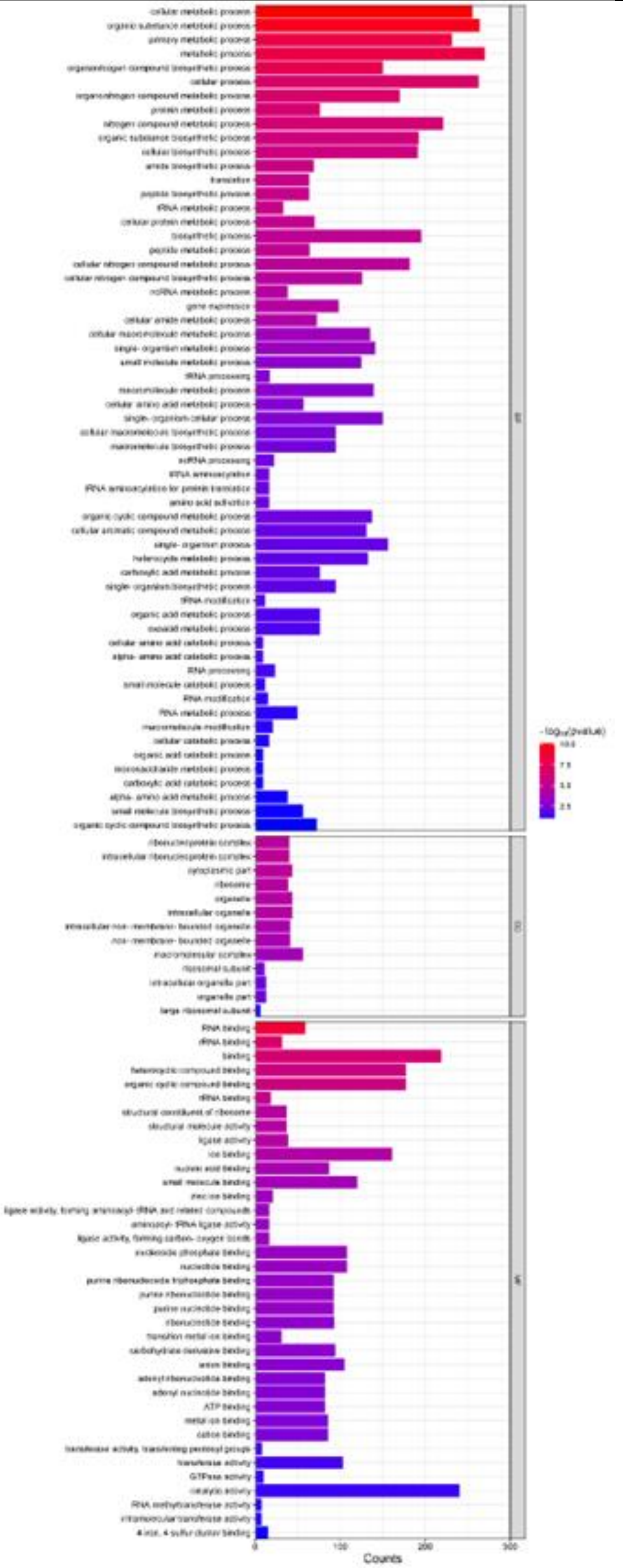

A. muciniphila JCM 30893

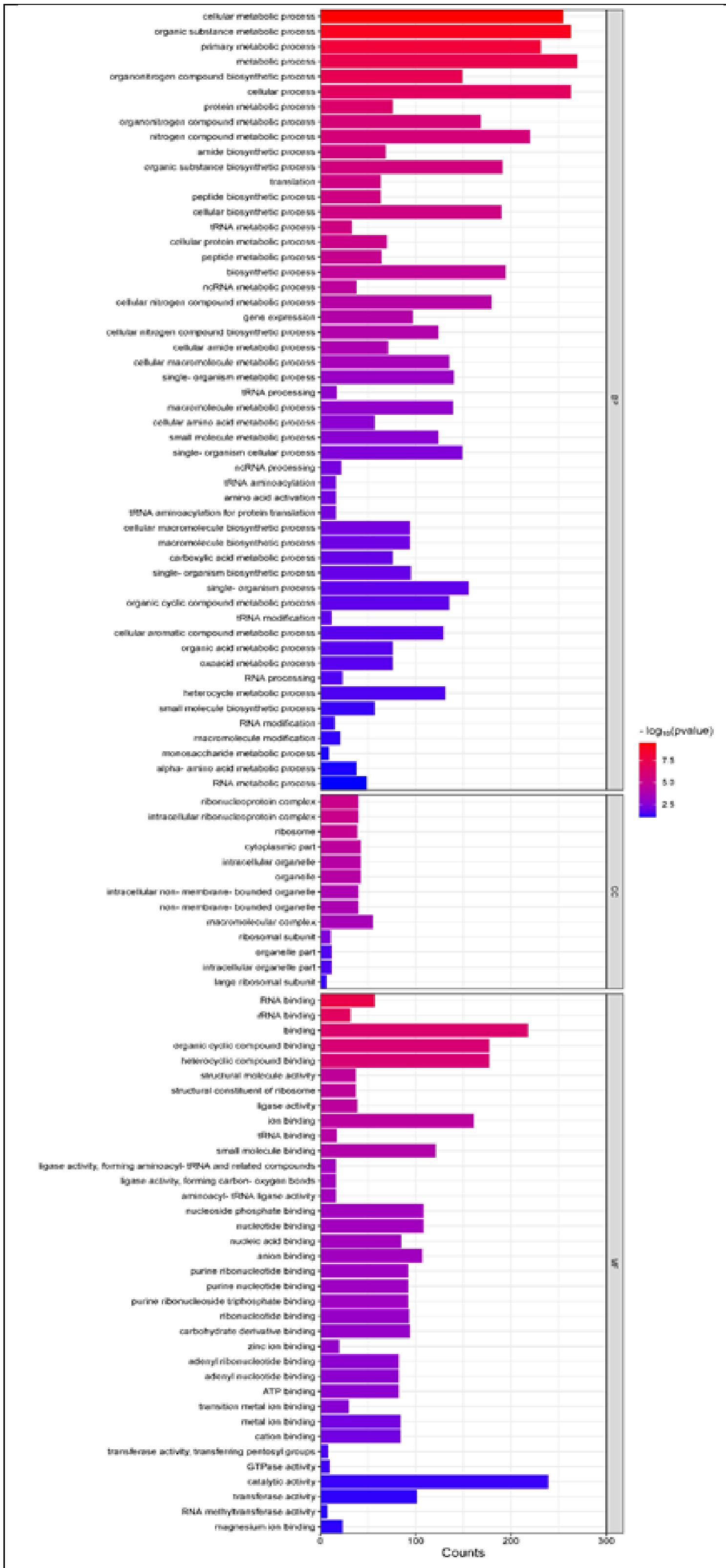

A. muciniphila KGMB01990

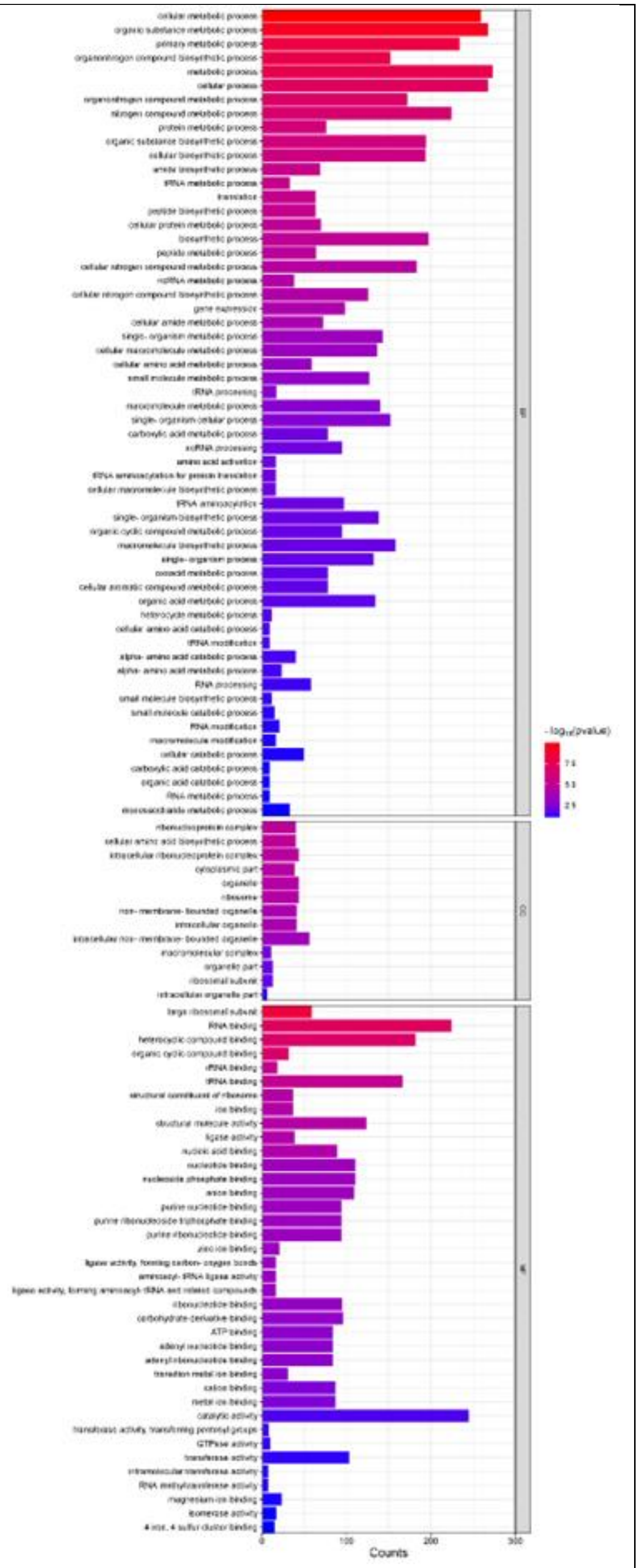

A. muciniphila MDA JAX AM001

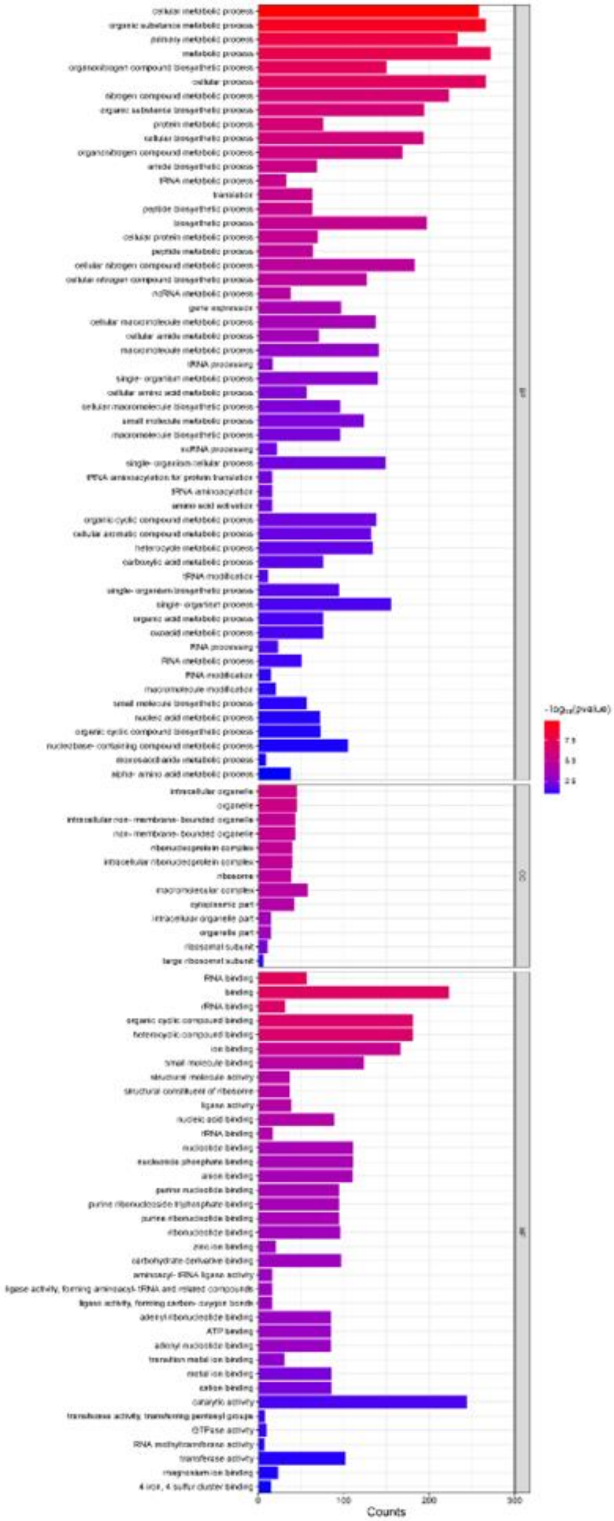	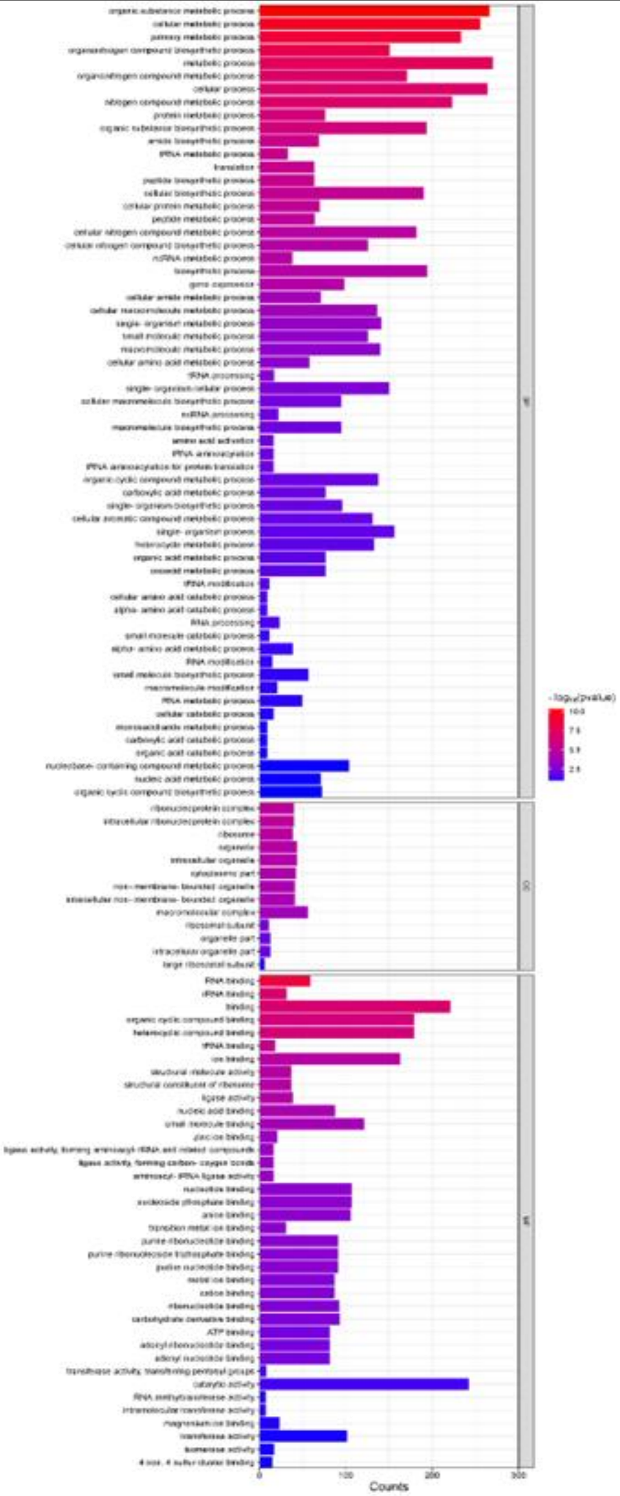
A. muciniphila OB2A FAA NM	*A. muciniphila YL44*

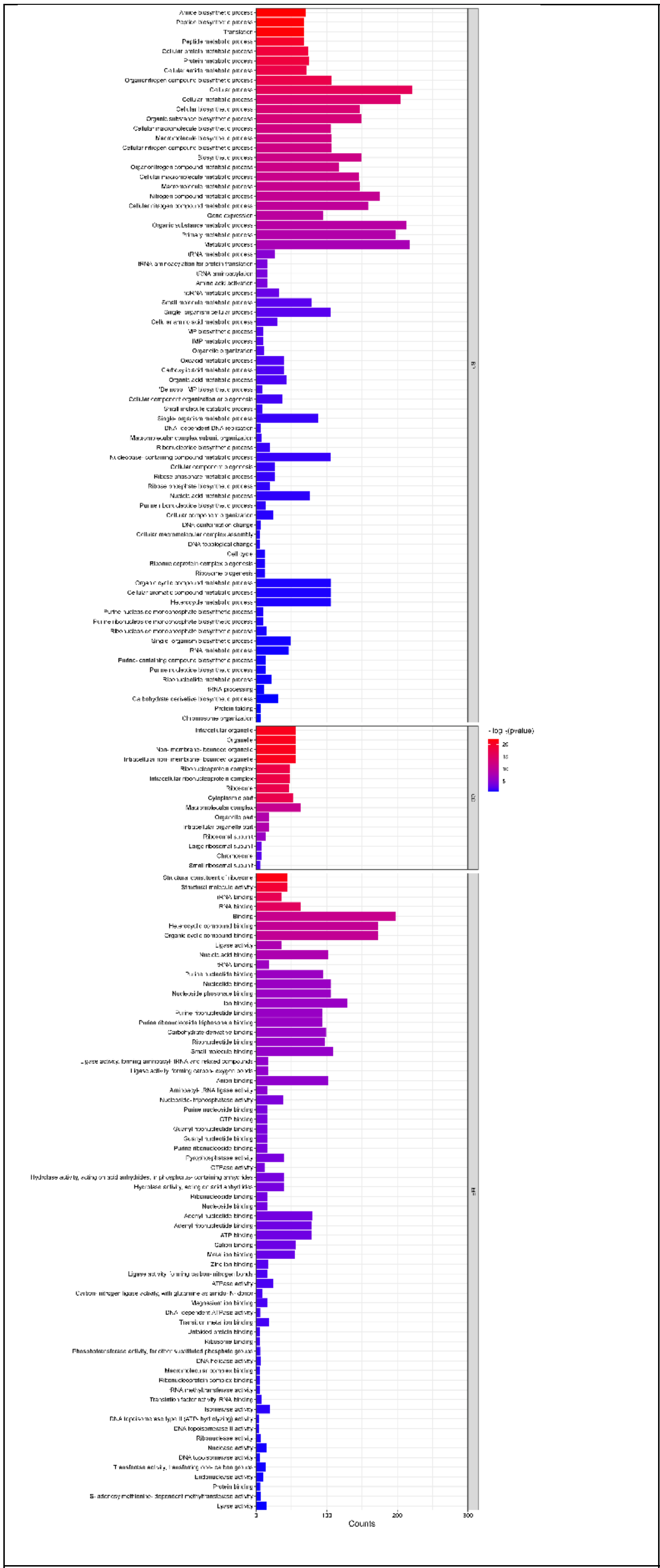

L. acidophillus DSM20079

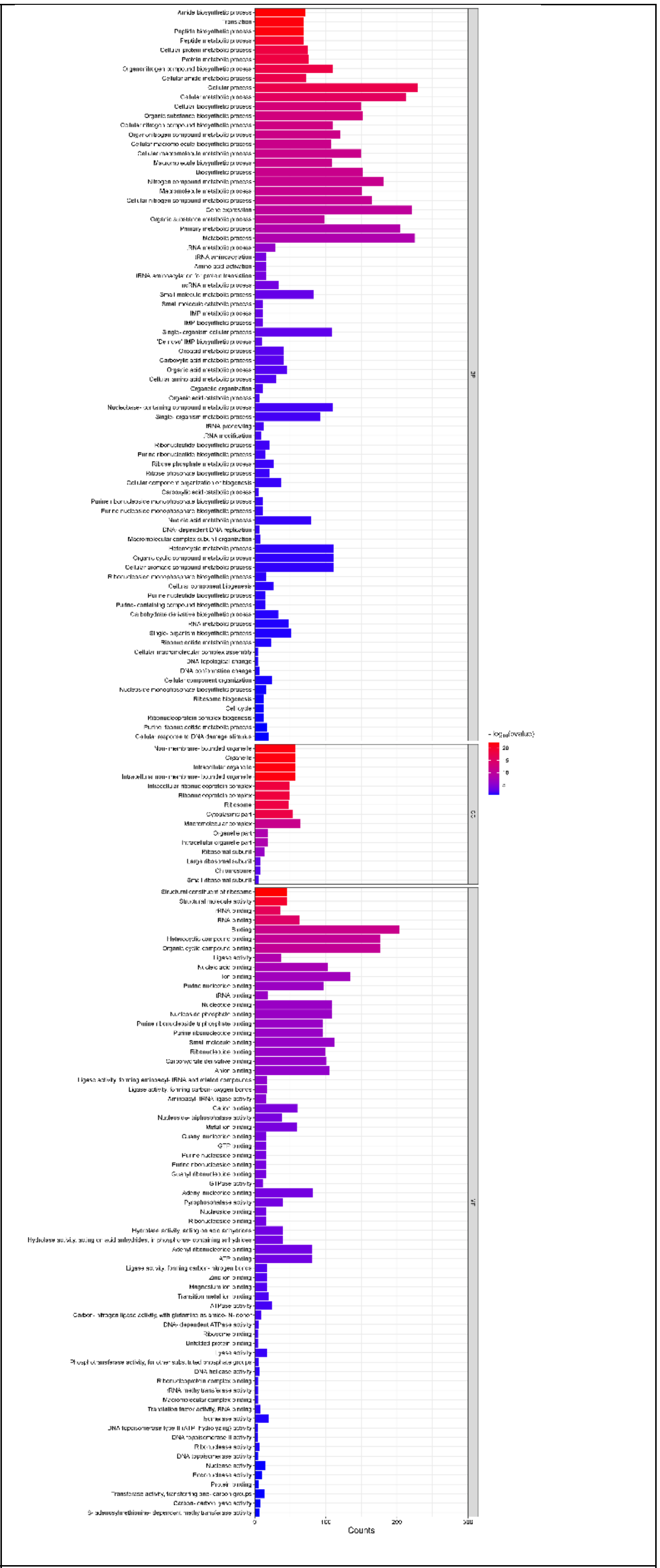

L. acidophillus W626

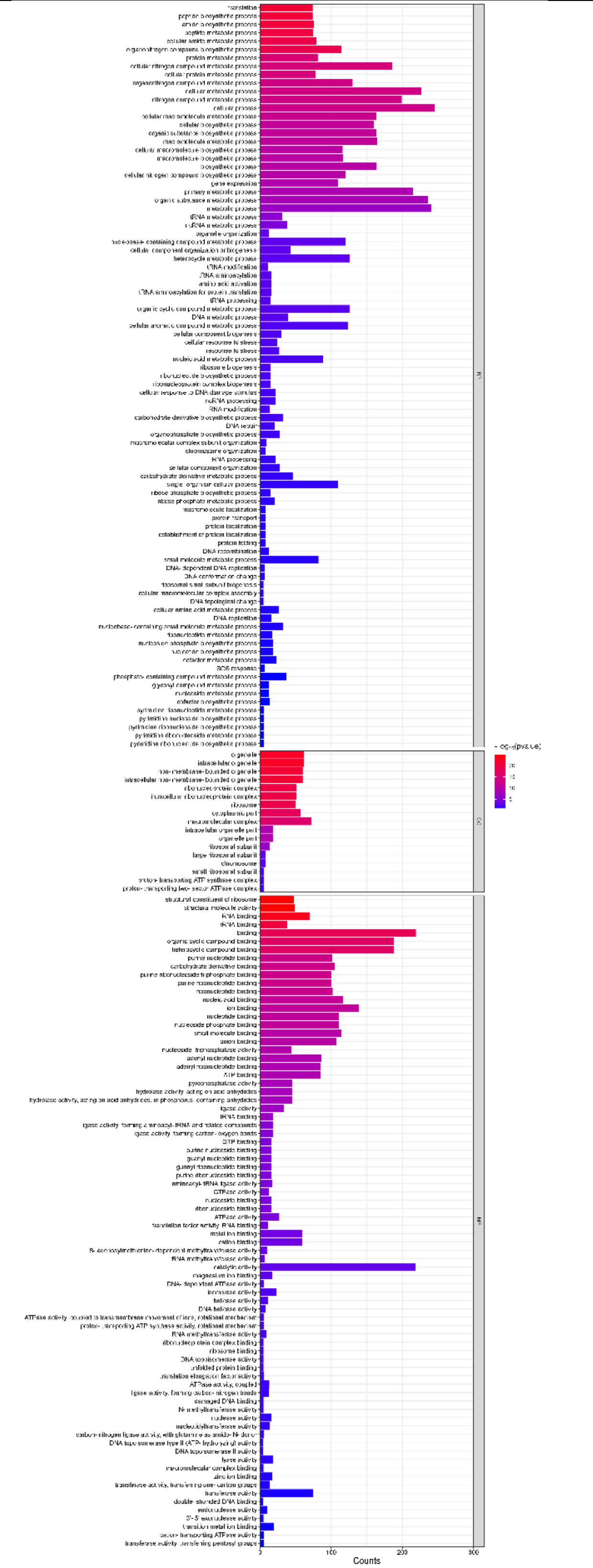

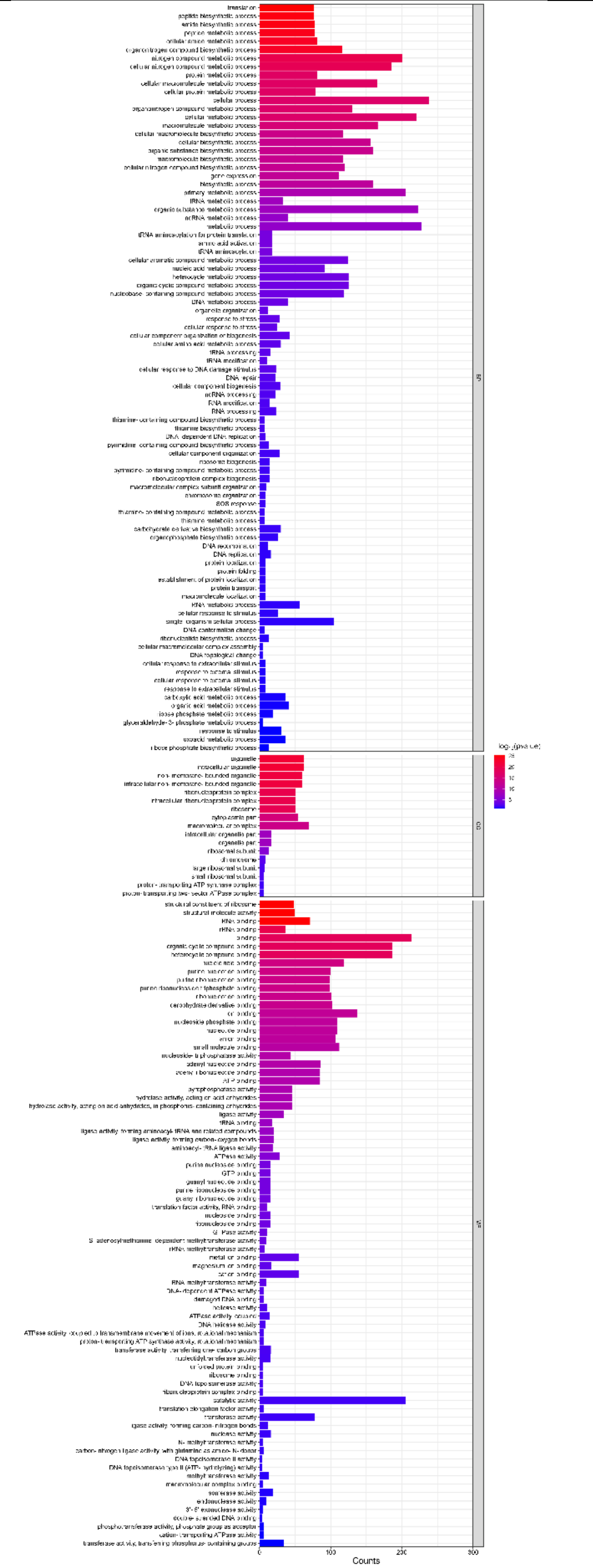

L. brevis BDGP6	*L. brevis CD0817*

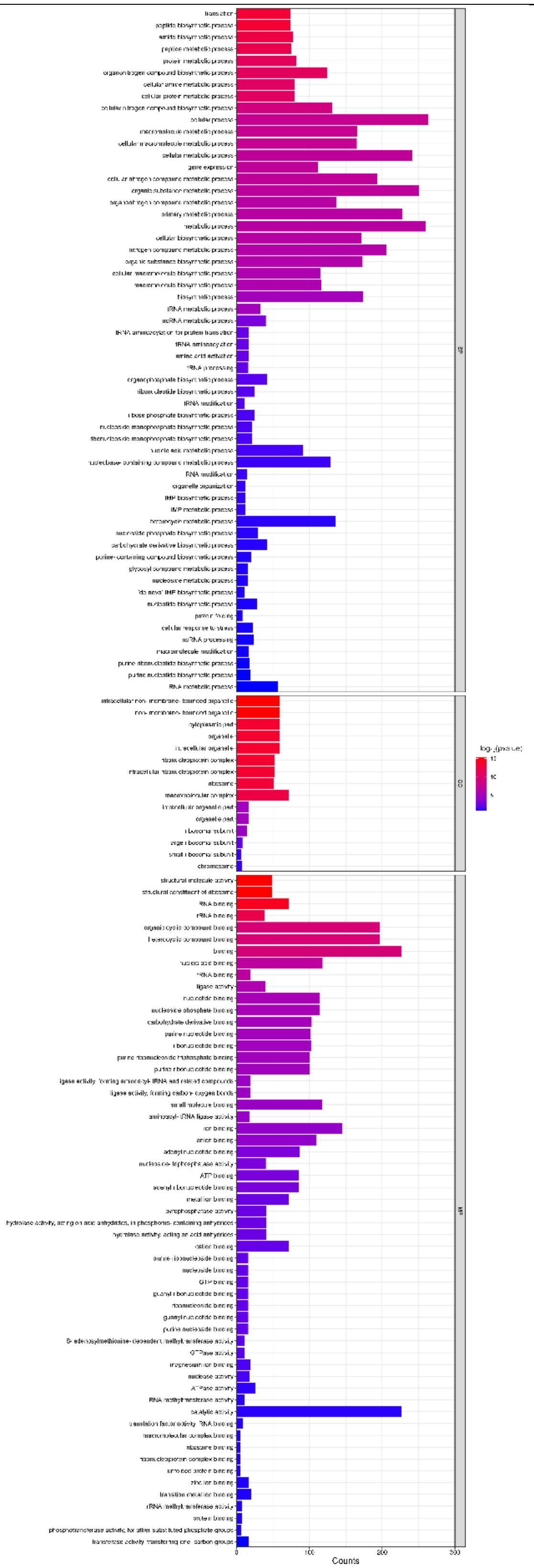

L. casei LC5

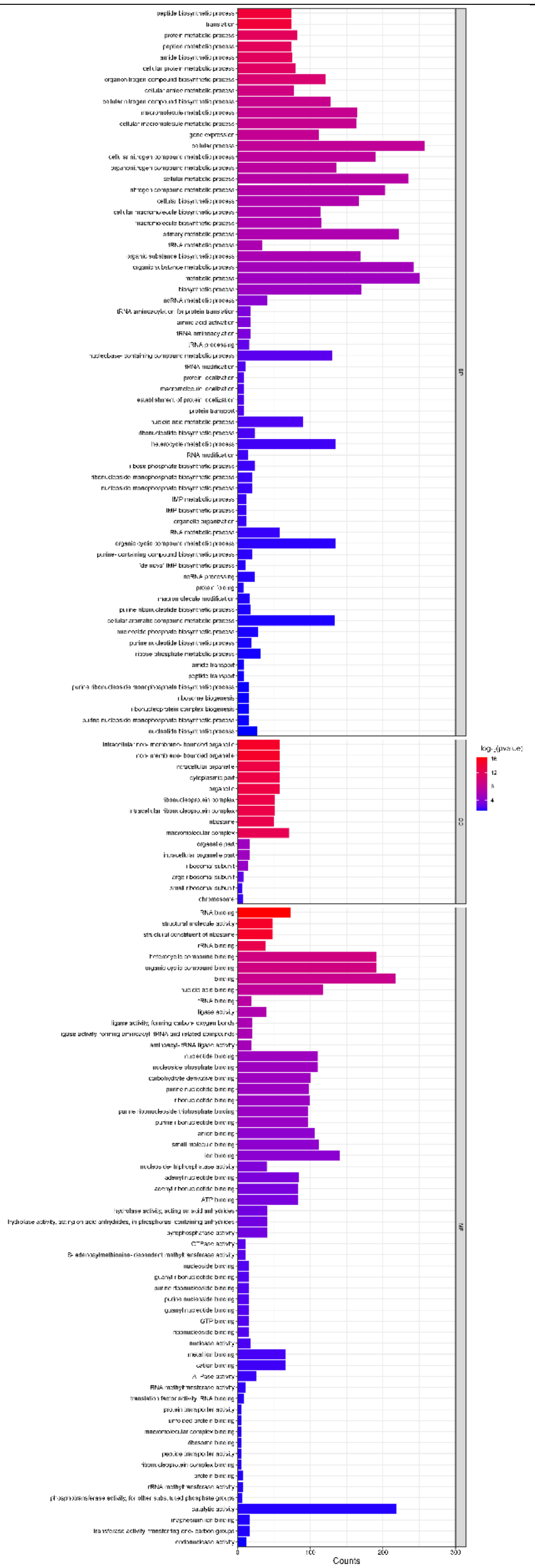

L. casei MGB0470

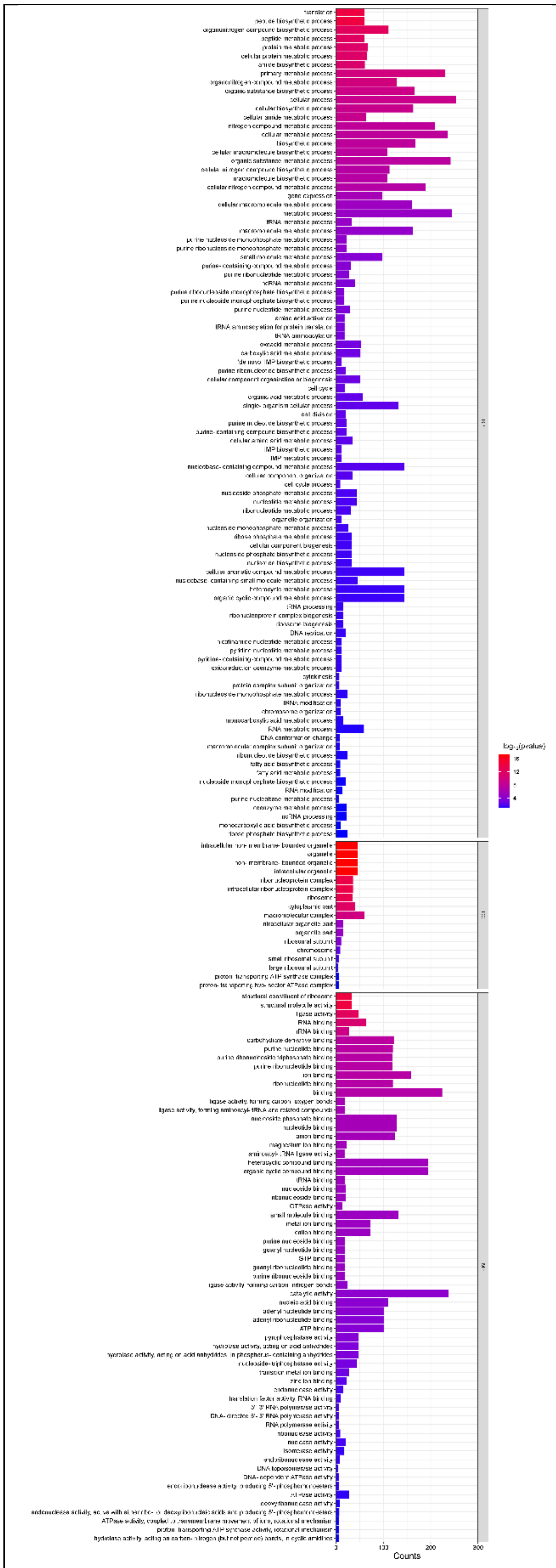	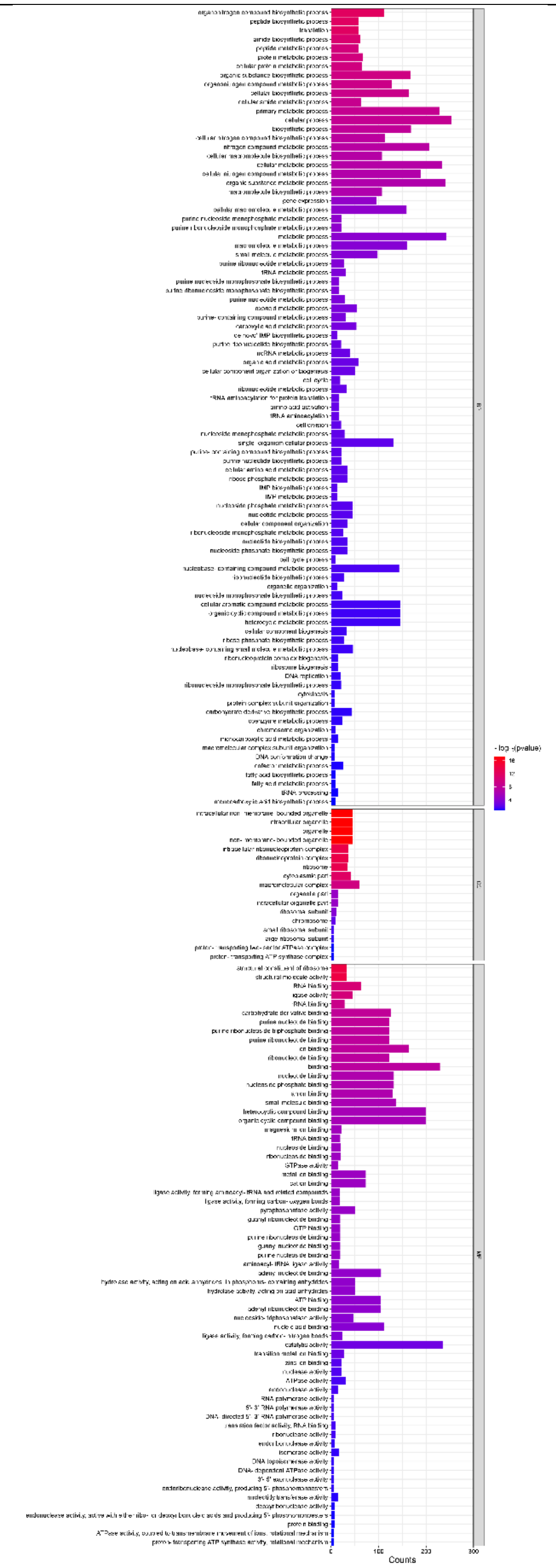
L. delbruckii bulgaricus MN-BM-F01	*L. delbruckii lactis KCCM-34717*

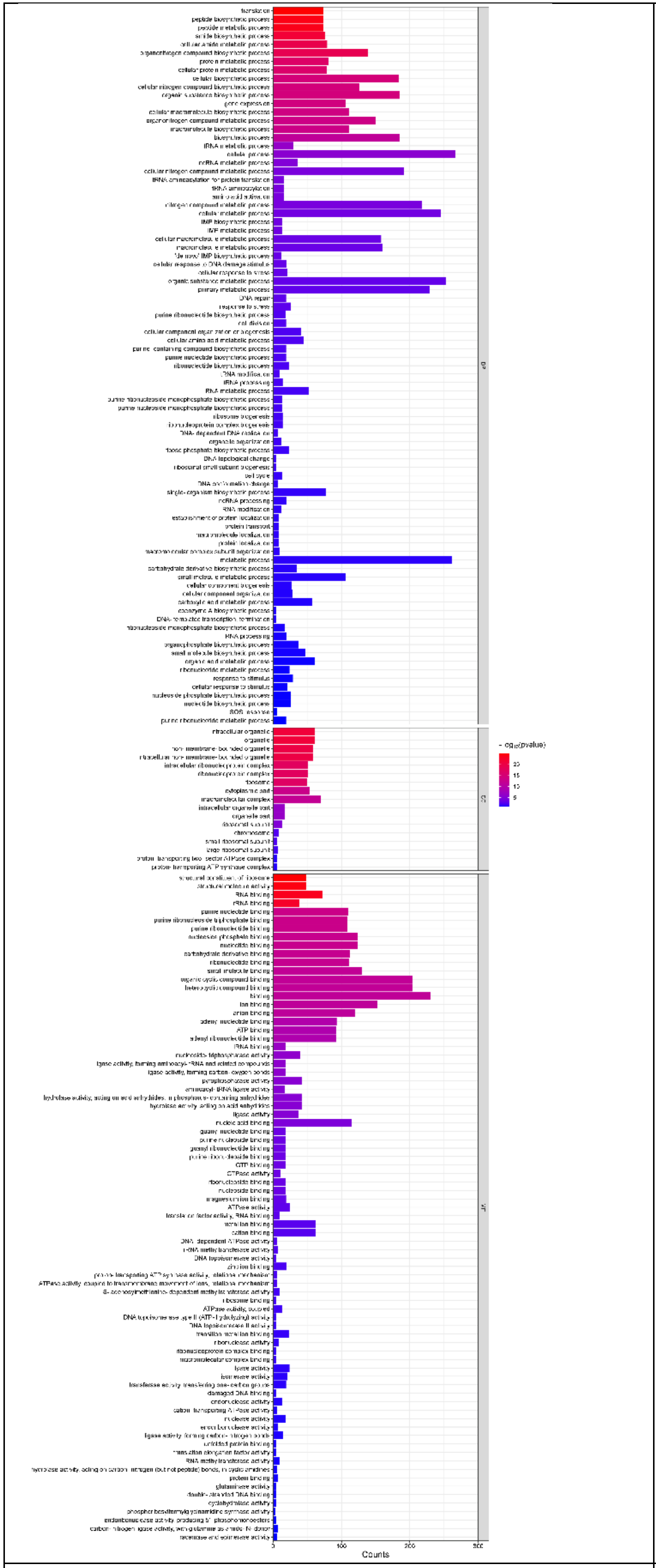	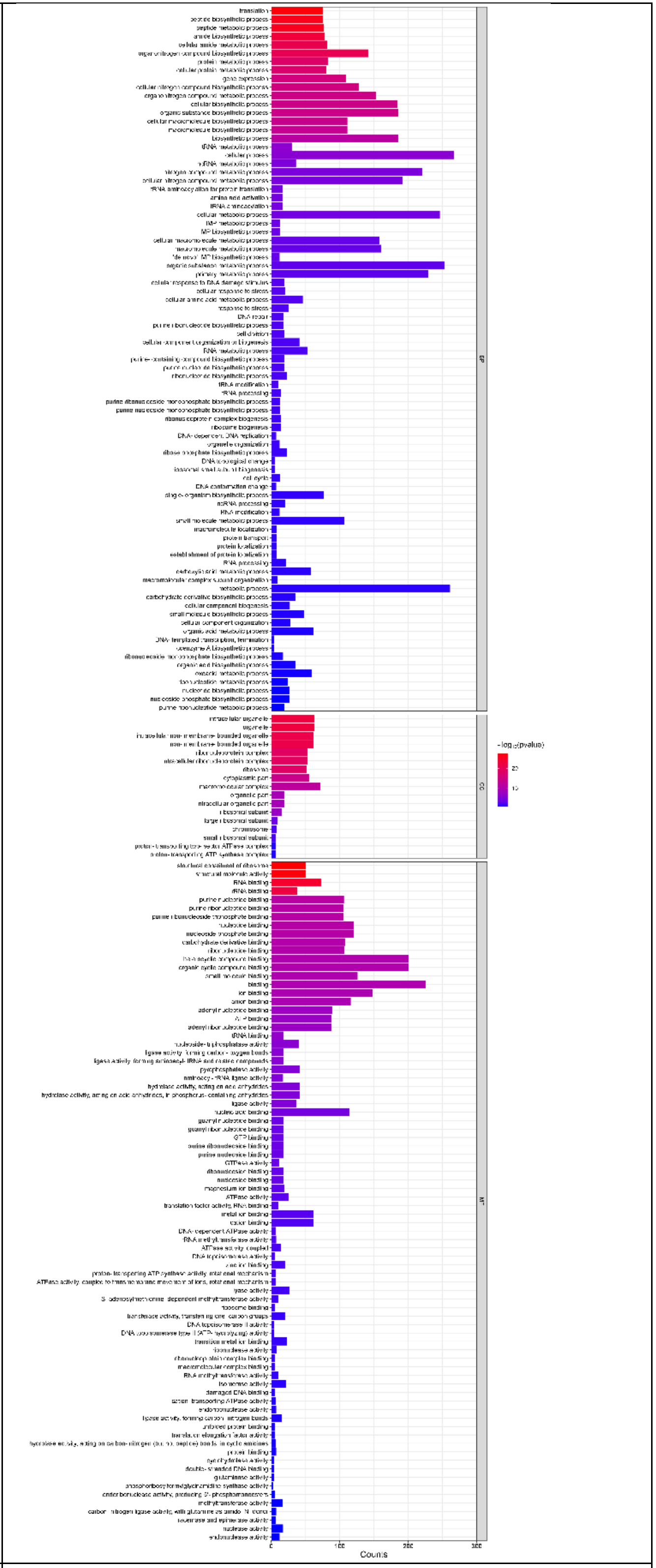
L. fermentum FTDC8312	*L. fermentum LMT2-75*

Sub-system Features Analysis of *Akkermansia muciniphila* and *Lactobacillus spp.* genomes

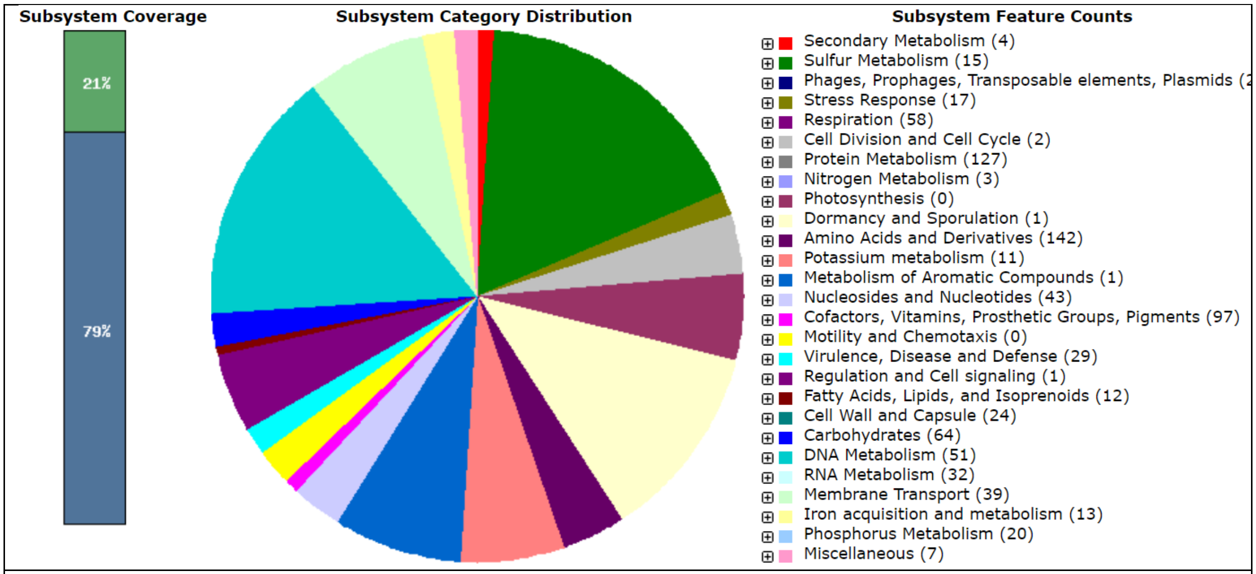

A. muciniphila AKK1370

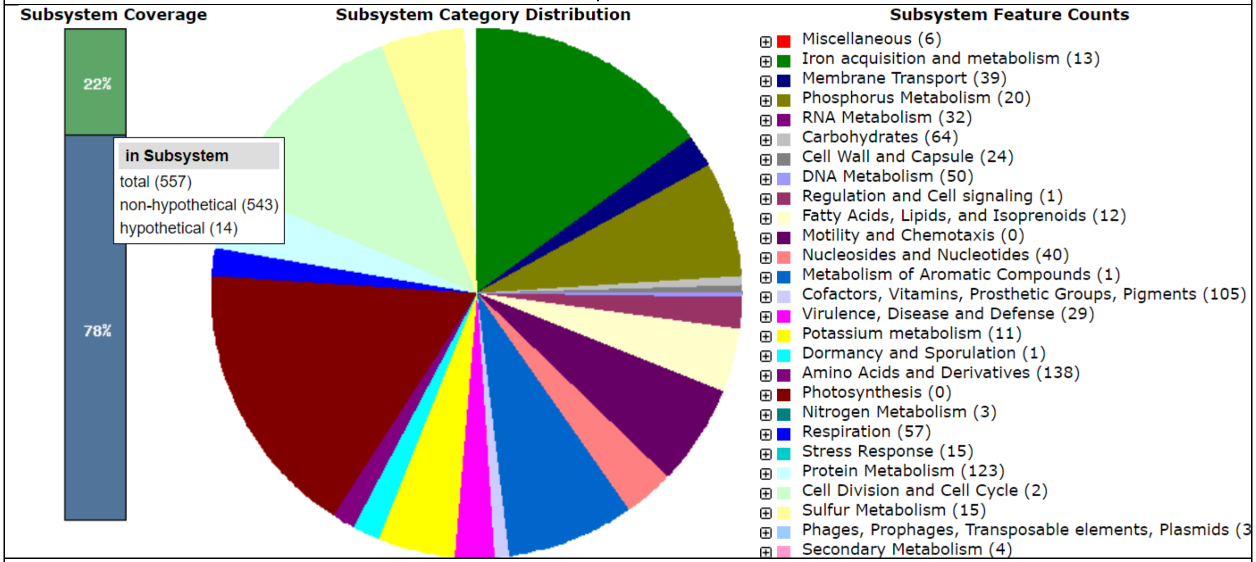

A. muciniphila AM06

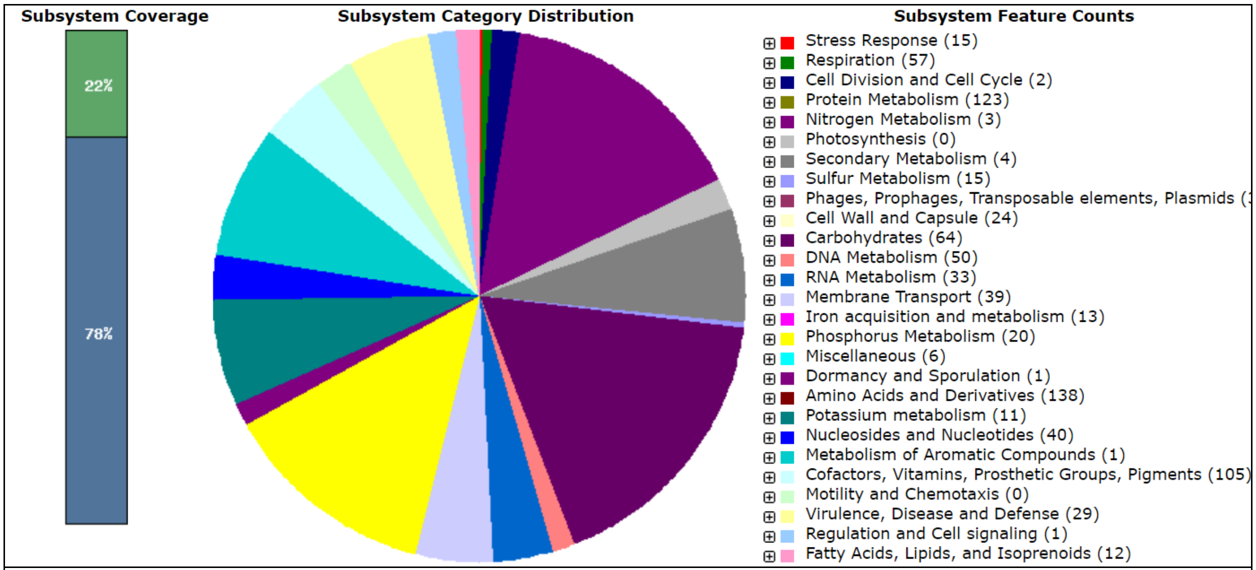

A. muciniphila ATCC BAA 835

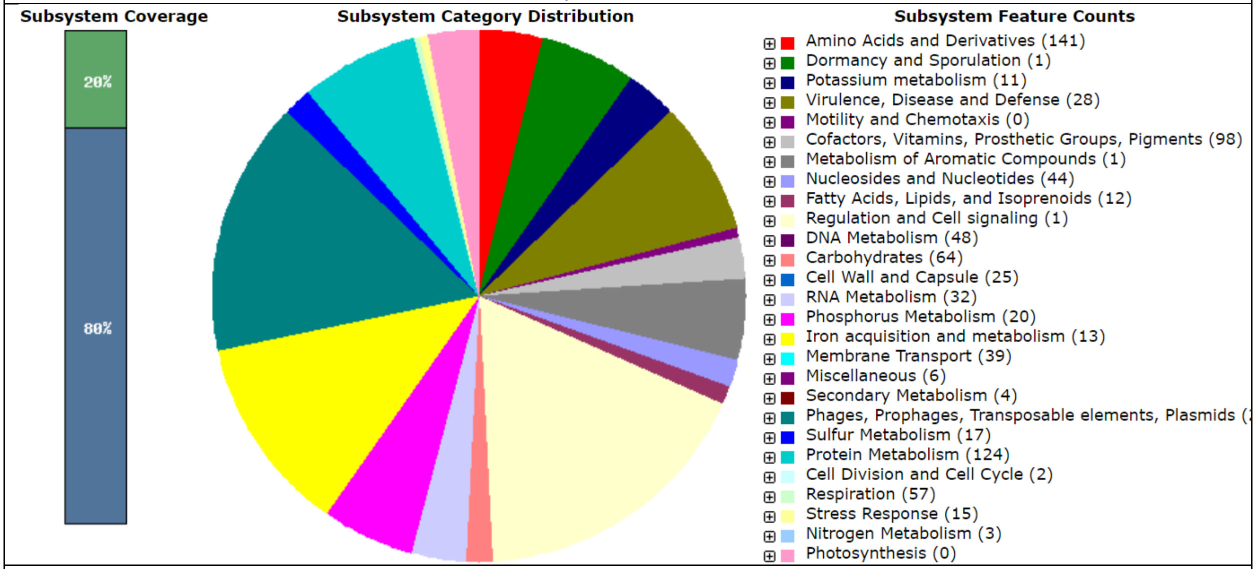

A. muciniphila CBA 5201

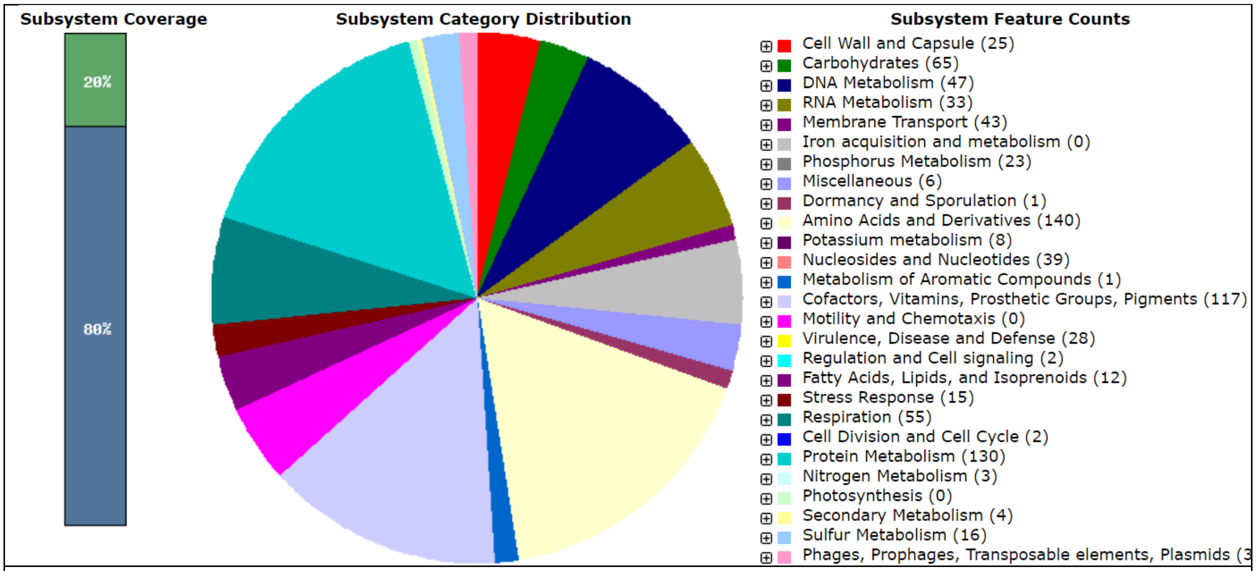

A. muciniphila EB AMDK 40

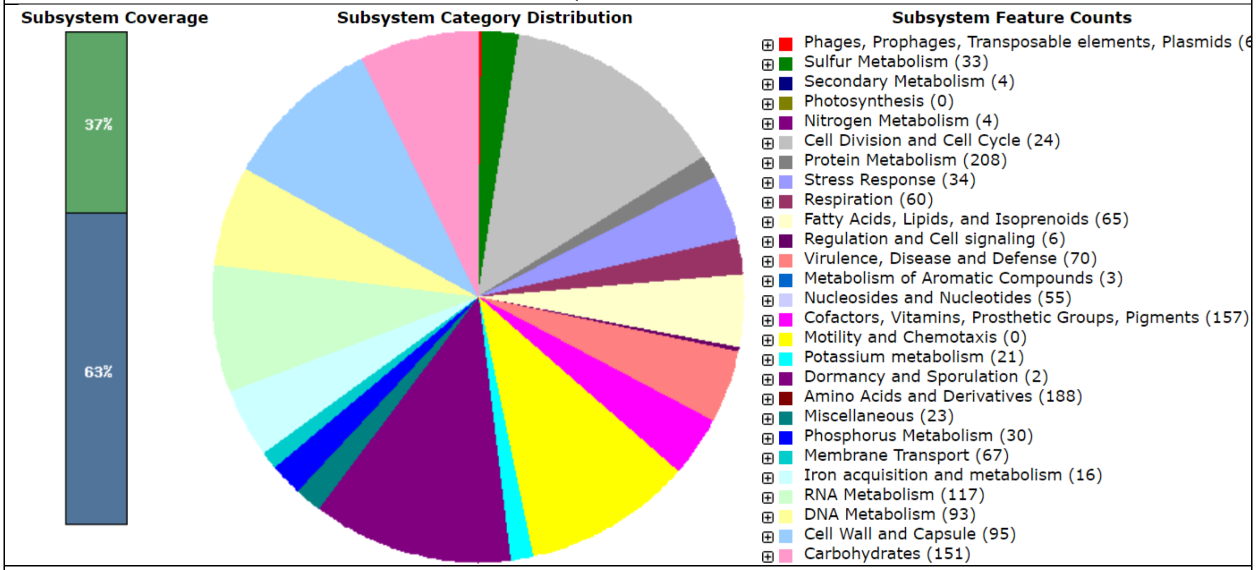

A. muciniphila JCM 30893

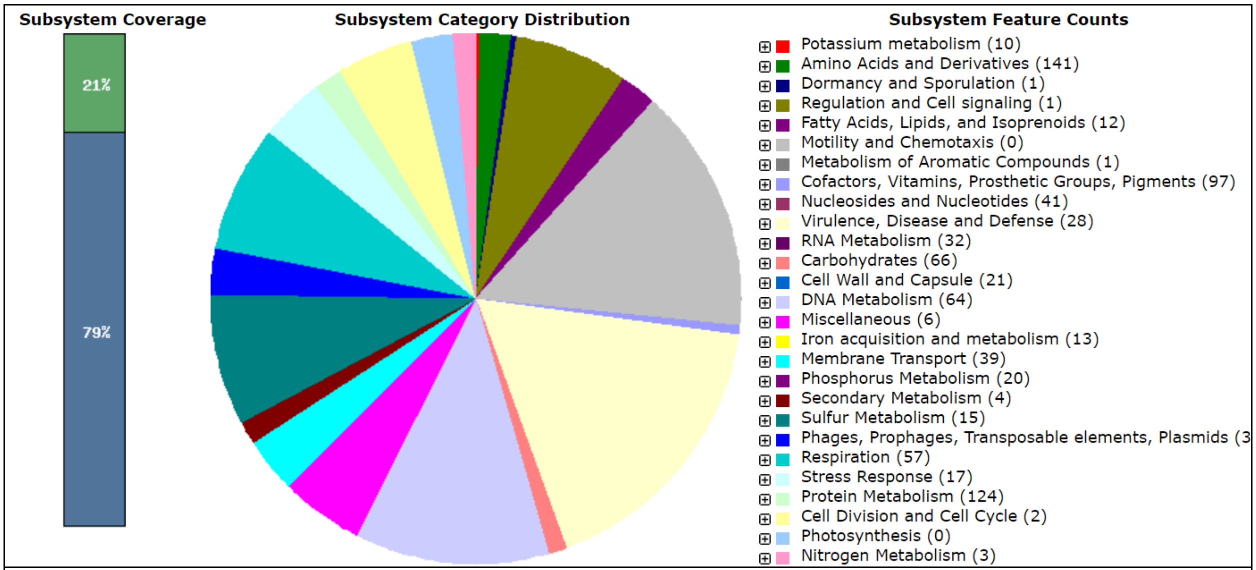

A. muciniphila KGMB01990

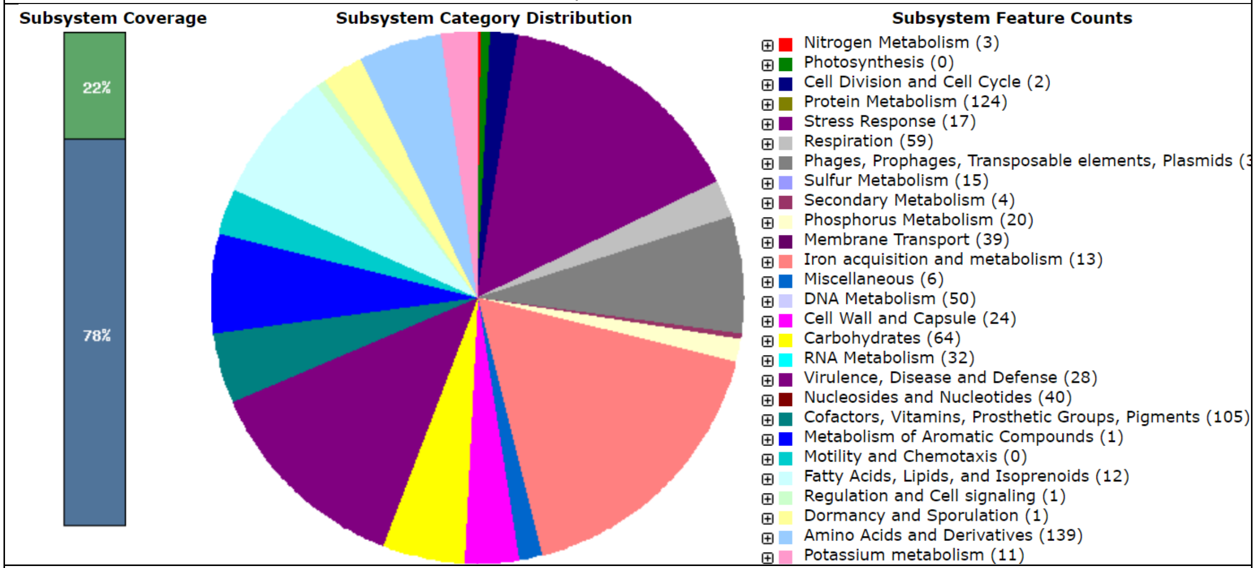

A. muciniphila MDA JAX AM001

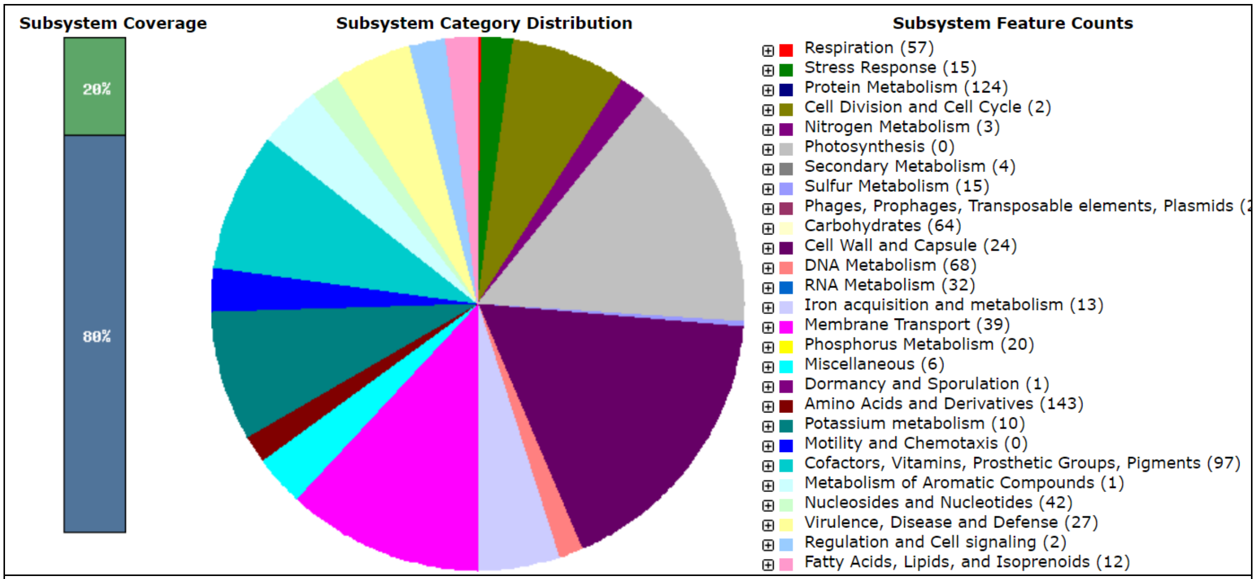

A. muciniphila OB2A FAA NM

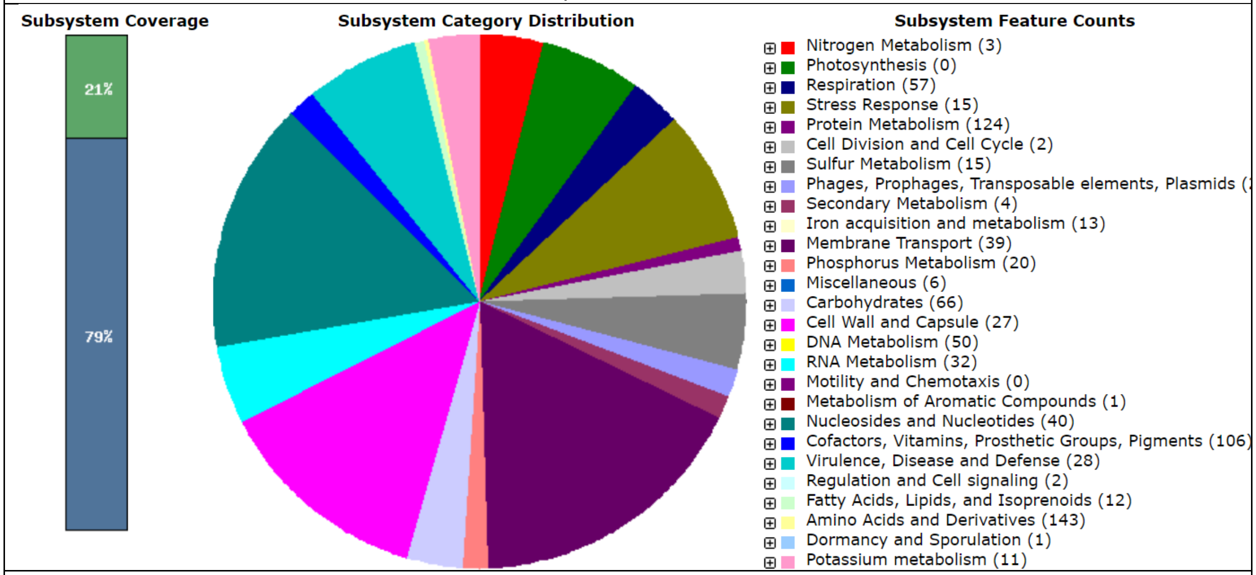

A. muciniphila YL44

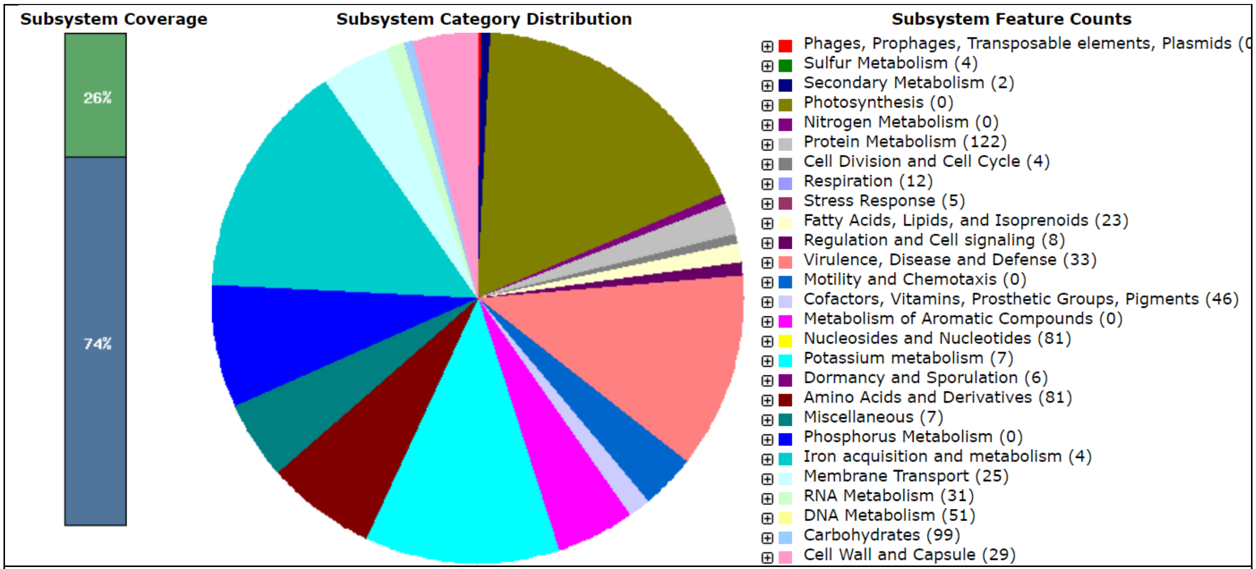

L. acidophillus DSM20079

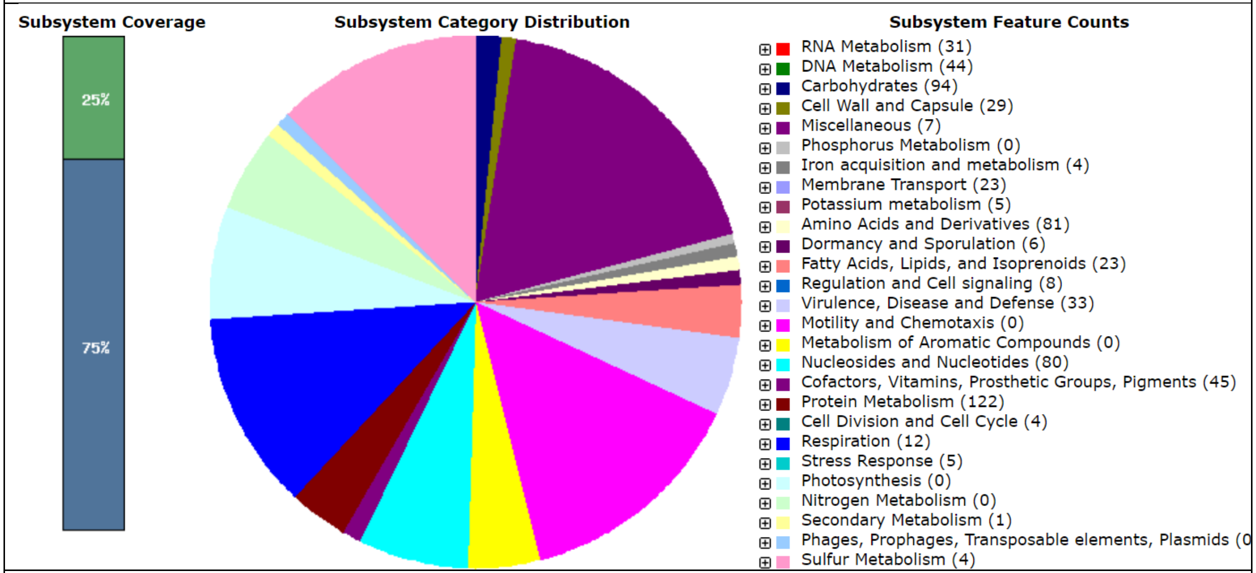

L. acidophillus W626

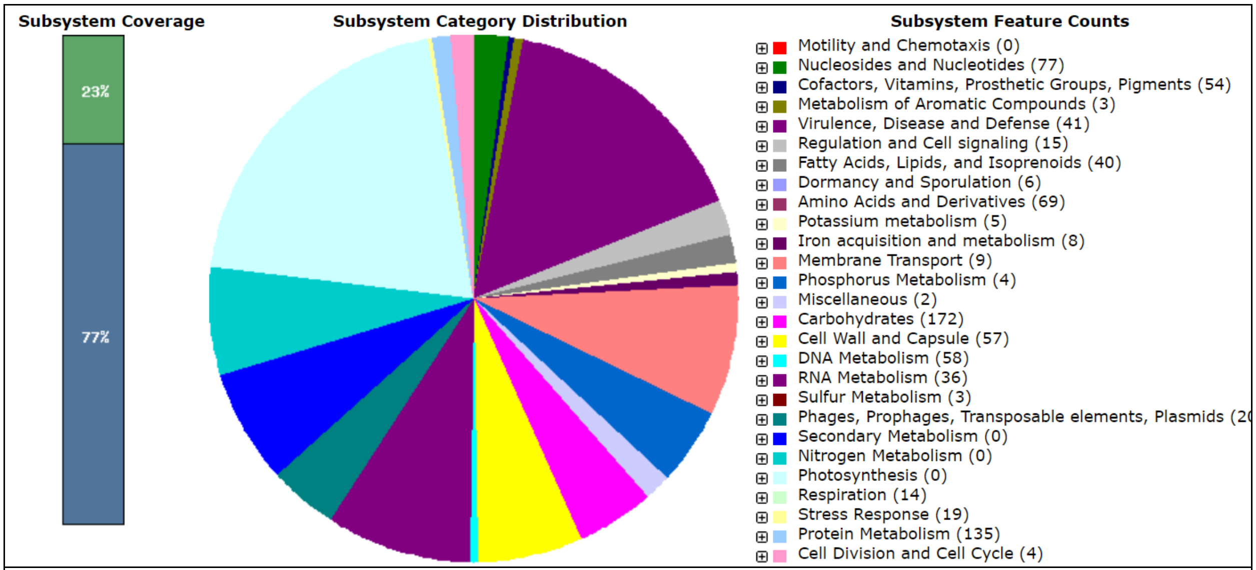

L. brevis BDGP6

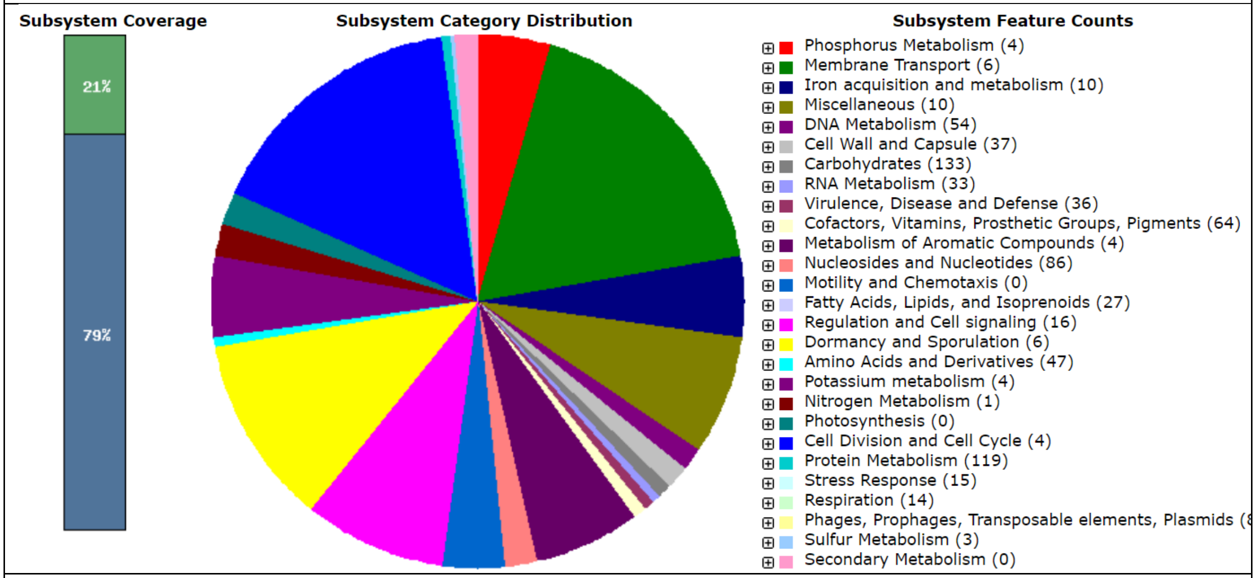

L. brevis CD0817

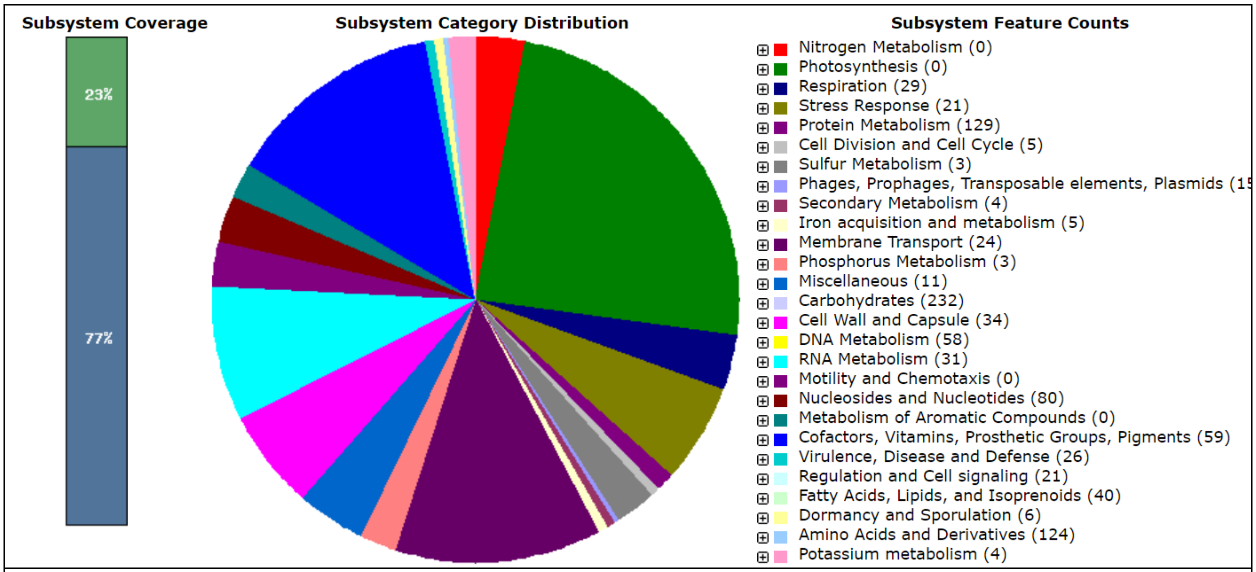

L. casei LC5

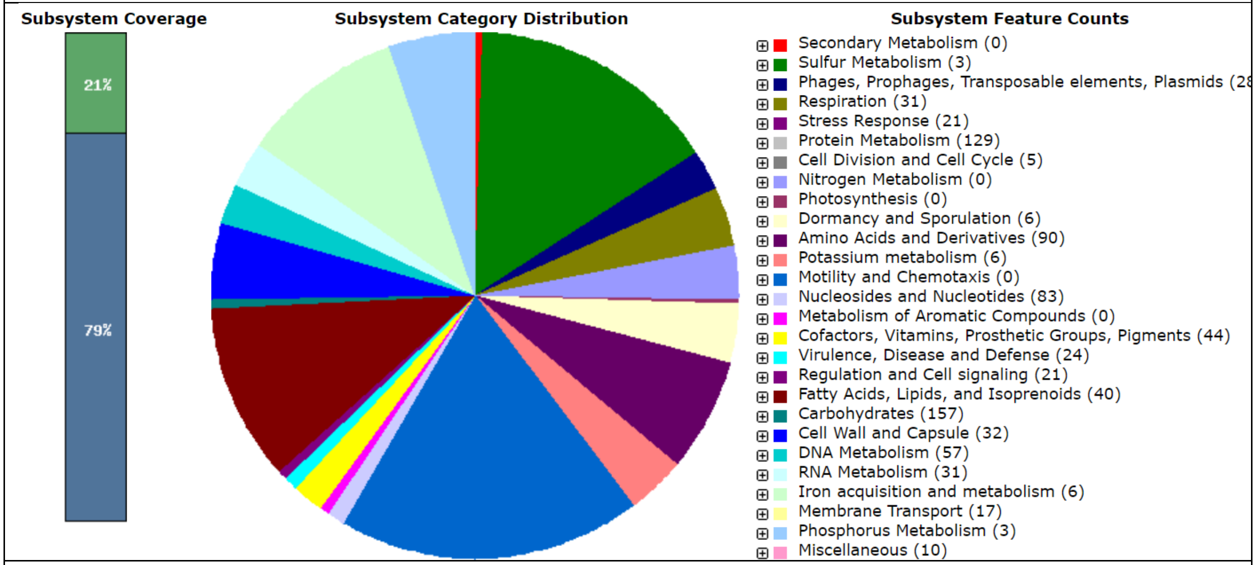

L. casei MGB0470

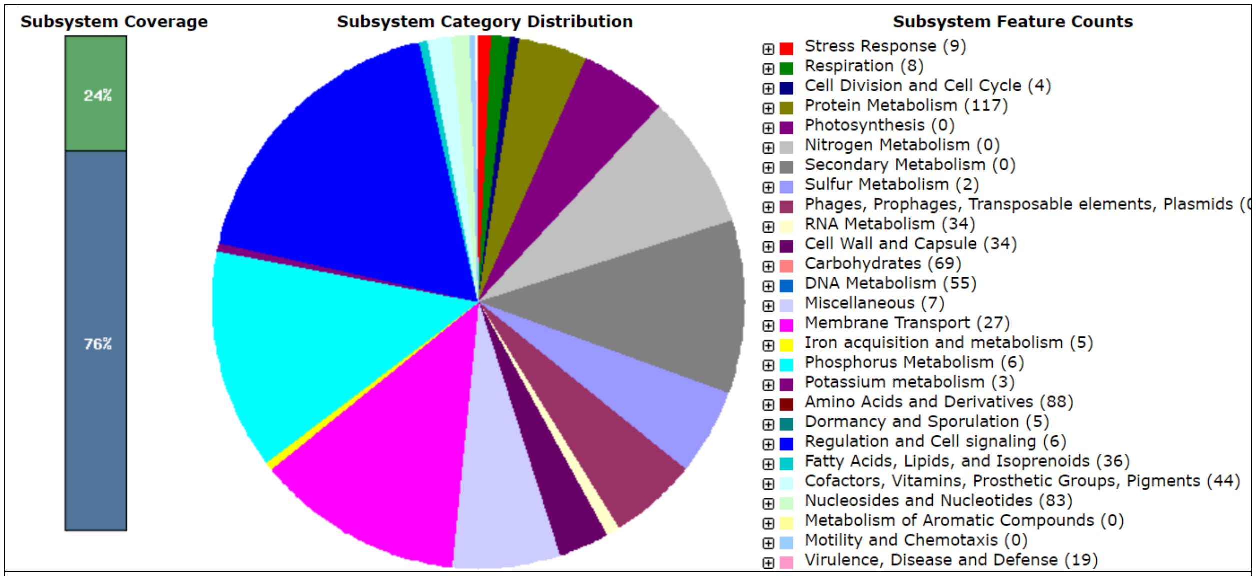

L. delbruckii bulgaricus MN-BM-F01

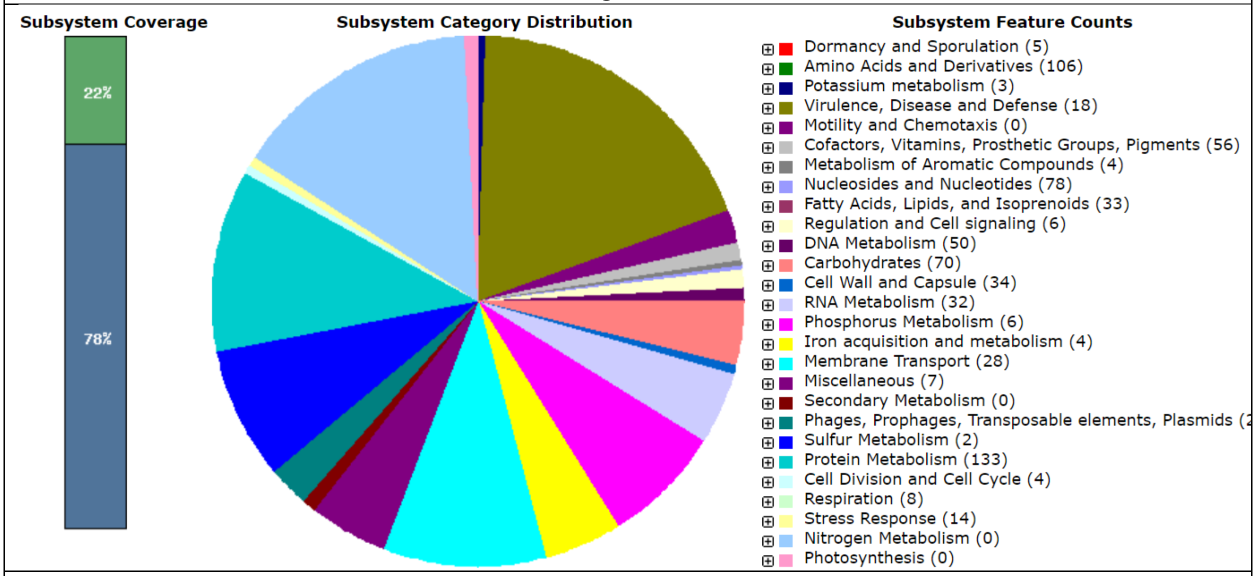

L. delbruckii lactis KCCM-34717

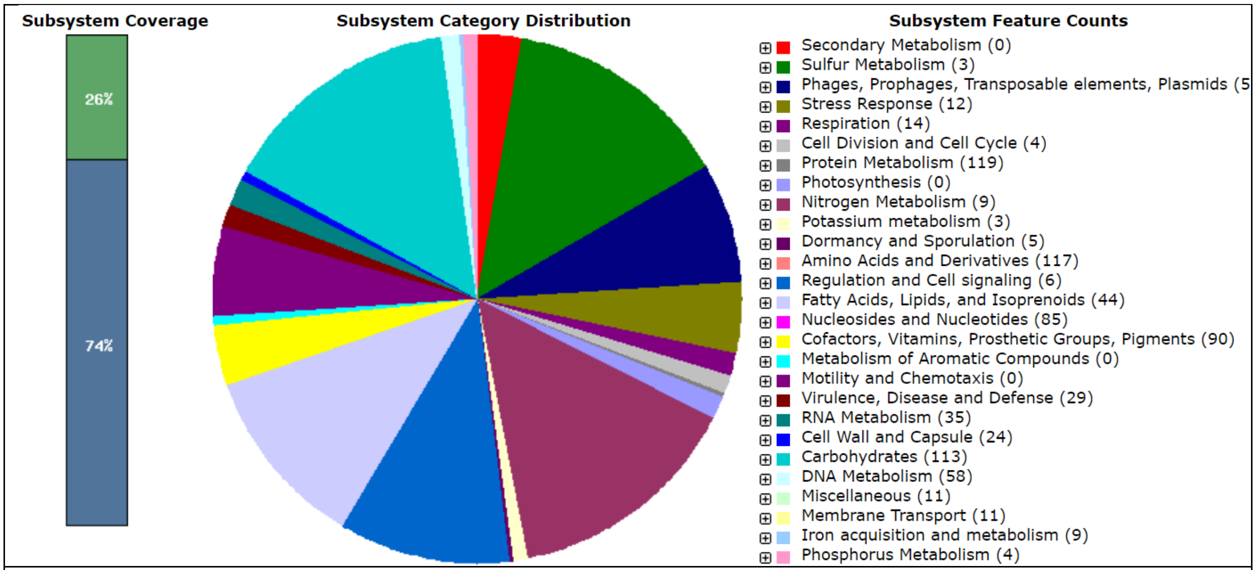

L. fermentum FTDC8312

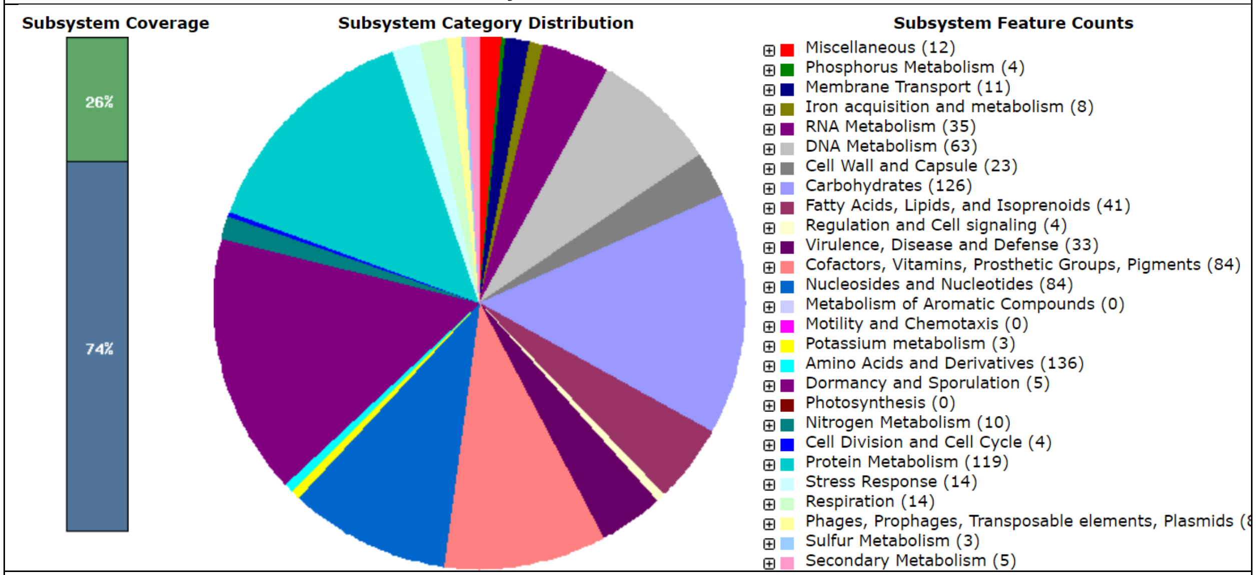

L. fermentum LMT2-75
